# A *Phytophthora sojae* effector suppresses endoplasmic reticulum stress-mediated immunity by stabilizing plant Binding immunoglobulin Proteins

**DOI:** 10.1038/ncomms11685

**Published:** 2016-06-03

**Authors:** Maofeng Jing, Baodian Guo, Haiyang Li, Bo Yang, Haonan Wang, Guanghui Kong, Yao Zhao, Huawei Xu, Yan Wang, Wenwu Ye, Suomeng Dong, Yongli Qiao, Brett M. Tyler, Wenbo Ma, Yuanchao Wang

**Affiliations:** 1 Department of Plant Pathology, Nanjing Agricultural University, 210095 Nanjing, China; 2 Key Laboratory of Integrated Management of Crop Diseases and Pests (Ministry of Education), 210095 Nanjing, China; 3 Institute of Crop Science, Chinese Academy of Agricultural Sciences, 100081 Beijing, China; 4 Center for Genome Research and Biocomputing and Department of Botany and Plant Pathology, Oregon State University, Corvallis, Oregon 97331, USA; 5 Department of Plant Pathology and Microbiology, University of California, Riverside, California 92521, USA; 6 Center for Plant Cell Biology, University of California, Riverside, California 92521, USA

## Abstract

*Phytophthora* pathogens secrete an array of specific effector proteins to manipulate host innate immunity to promote pathogen colonization. However, little is known about the host targets of effectors and the specific mechanisms by which effectors increase susceptibility. Here we report that the soybean pathogen *Phytophthora sojae* uses an essential effector PsAvh262 to stabilize endoplasmic reticulum (ER)-luminal binding immunoglobulin proteins (BiPs), which act as negative regulators of plant resistance to *Phytophthora*. By stabilizing BiPs, PsAvh262 suppresses ER stress-triggered cell death and facilitates *Phytophthora* infection. The direct targeting of ER stress regulators may represent a common mechanism of host manipulation by microbes.

## Abstract (web summary)

*Phytophthora* pathogens manipulate host plant immune responses by secreting effector proteins into plant cells. Here, Jing *et al*. show that the *Phytophthora sojae* Avh262 effector negatively regulates plant resistance by binding to host BiP proteins and suppressing ER-stress induced cell death

## Graphical abstract

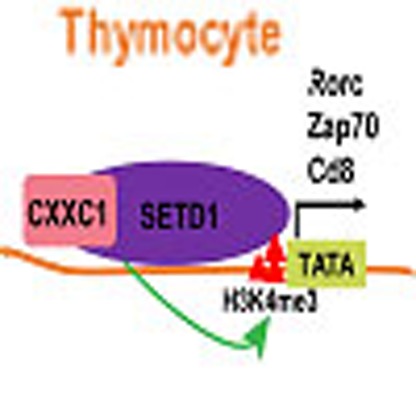

Plants have evolved a two-layer surveillance system to protect themselves against pathogens. In one layer, plant basal defence responses are activated upon the perception of pathogen-associated molecular patterns (PAMP) by pattern recognition receptors at the cell surface, resulting in PAMP-triggered immunity (PTI) that prevents infection by most microbes^1^. However, successful pathogens deliver effectors into plant cells to suppress PTI and establish parasitism^1^. In response, plants evolved a second layer of immunity, called effector-triggered immunity (ETI), that is initiated upon recognition of effectors by specific intracellular receptors^1^. Pathogen effectors can also attenuate ETI^2^,^3^,^4^,^5^,^6^. Thus, research on effector functions has not only elucidated the pathogenic mechanisms of pathogens, but also has identified novel components of plant immune systems.

Fungi and oomycetes, are among the most damaging pathogens to agriculture. For example, *Phytophthora sojae* (*P. sojae*) is one of the most important pathogens of soybean. Oomycetes secrete RxLR effectors that are translocated into plant cells during infection^3^,^7^. The conserved N-terminal RxLR motif is required to mediate the delivery of these virulence proteins into host cells, probably via specific infection structures called haustoria and directly from hyphae^2^,^3^. *Phytophthora* genomes each encode about 300–700 RxLR effectors^8^,^9^,^10^,^11^. RxLR effectors of *P. sojae* suppress plant immunity via transcriptional programming and functional cooperation^12^. Many *Phytophthora* RxLR effectors can interfere with plant immunity by modifying host targets. For example, *Phytophthora infestans* (*P. infestans*) Avrblb2 focally accumulates near haustoria and blocks the secretion of the plant papain-like cysteine protease C14 into the apoplast^13^. *P. sojae* Avr3b functions as an ADP-ribose/NADH pyrophosphorylase and promotes virulence by its enzyme activity^14^. Two *P. sojae* RxLR effectors, PSR1 and PSR2, suppress RNA silencing in plants by inhibiting the biogenesis of small RNAs^15^. To date, the plant targets and molecular mechanisms of the vast majority of RxLR effectors are still unknown.

The endoplasmic reticulum (ER) is a membrane-bound compartment that mediates cellular processes such as calcium homoeostasis and protein processing^16^,^17^. In the ER, proteins that are improperly folded or assembled are recognized by the ER quality control (ERQC) system and transported into the cytoplasm for ER-associated degradation (ERAD)^18^,^19^. The ERQC system consists of three pathways, one of which relies on the binding immunoglobulin protein (BiP) complex^20^. As the most abundant ER chaperones and key components of the ERQC machinery, BiPs play an important role in the unfolded protein response (UPR) by regulating stress transducers, such as the activating transcription factor 6 (ATF6), protein kinase RNA-like ER kinase (PERK) and inositol-requiring enzyme 1 (IRE1)^21^,^22^.

Emerging evidence indicates that ER stress-related cell death is associated with microbe infection. For example, the endophytic fungus *Piriformospora indica* activates ER stress-mediated cell death by inhibiting the UPR-related pro-survival machinery^23^. Thus ER-stress pathways could be targeted by pathogens to facilitate infection^24^. BiPs can regulate plant responses to abiotic and biotic stresses. For example, overexpression of *GmBiP4* in soybean (*Glycine max*) inhibited the UPR and increased tolerance to water loss^25^, and suppressed cell death associated with senescence and osmotic stress^26^, but at the same time intensified cell death and induction of SA-mediated responses triggered by avirulent *Pseudomonas* species^26^. AtBiP2 of *Arabidopsis thaliana* is involved in folding and secretion of pathogenesis-related (PR) proteins during systemic acquired resistance (SAR), as loss-of-function mutants of *AtBiP2* are defective in salicylic acid-elicited PR1 protein secretion^27^. Overexpression of a *Nicotiana benthamiana BiP* gene eliminated the triple gene block protein 3 (TGBp3)-induced hypersensitive response (HR), which is consistent with the cyto-protective role of the NbBiP in virus-infected leaves of *N. benthamiana*^28^.

Here we report the identification of BiPs as novel targets of a *P. sojae* RxLR effector, PsAvh262 that is essential for *P. sojae* infection. Ectopic expression of BiPs in both soybean hairy roots and *N. benthamiana* leaves enhanced susceptibility, suggesting that BiPs negatively regulate plant defence against *Phytophthora* infection. Our results suggest that PsAvh262 may promote infection by binding and stabilizing BiPs, resulting in attenuated plant defence responses.

## Results

### PsAvh262 is required for full virulence of *P. sojae*

**Figure 1:**
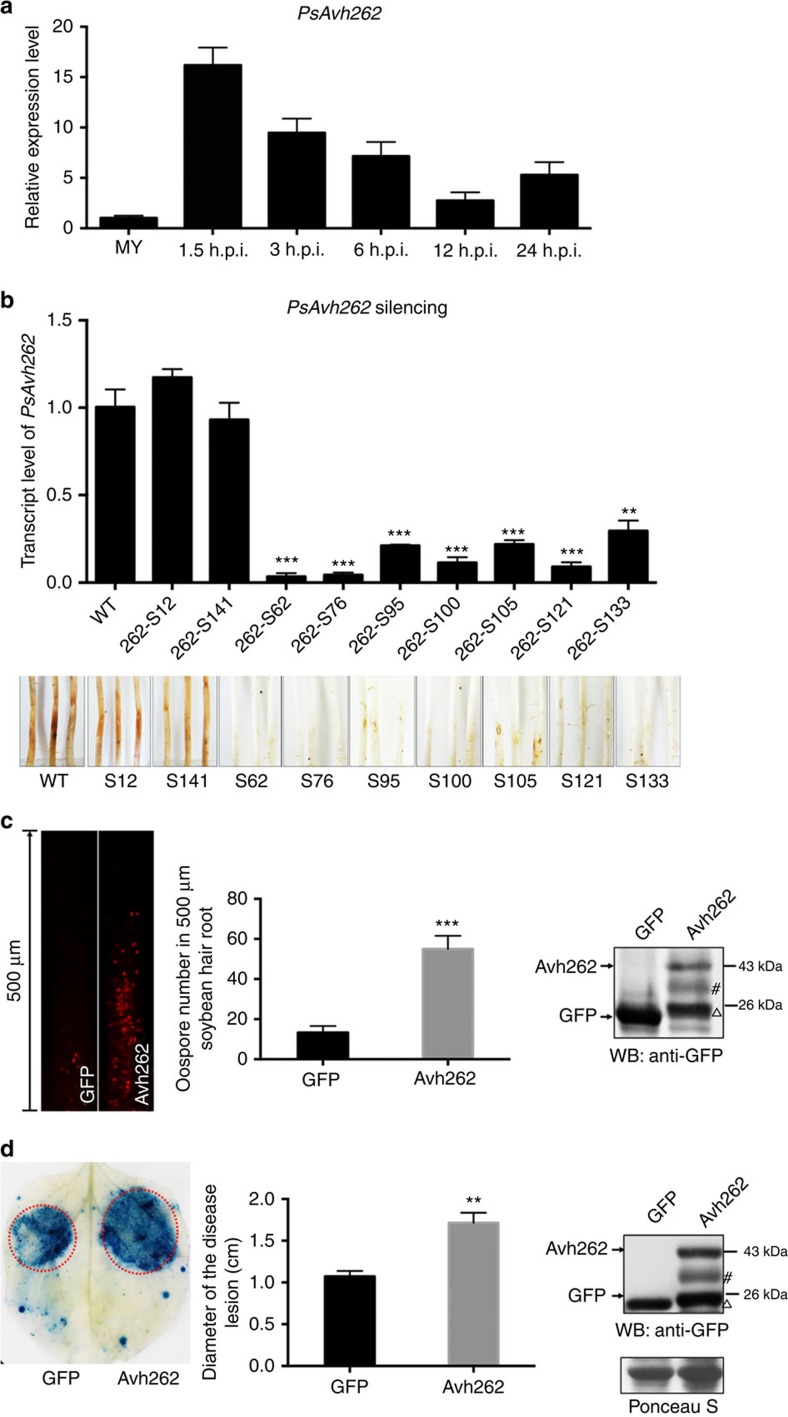
PsAvh262 is an essential virulence factor of *Phytophthora sojae*. (**a**) Expression profile of *PsAvh262* during *P. sojae* strain P6497 infection of soybean hypocotyls. The susceptible soybean cultivar Williams was used as the host. Total RNA was extracted from mycelia (MY) or infected soybean leaves at 1.5, 3, 6, 12 and 24 h post inoculation (h.p.i.). Transcript levels of PsAvh262 were determined by qRT–PCR. The *P. sojae actin* gene (VMD GeneID: 108986) was used as the pathogen internal control gene, (**b**) Silencing of *PsAvh262* in *P. sojae* greatly impaired the virulence in soybean hypocotyls. Relative transcript levels of *PsAvh262* (upper panel) in the *P. sojae* transformants were determined by qRT–PCR. Disease symptoms (lower panel) in etiolated hypocotyls were observed. Pictures were taken at 7 days post inoculation (d.p.i.). S12 and S141 were non-silenced transformants carrying the same silencing construct. (**c**) Expression of PsAvh262 in soybean hairy roots enhanced *P. sojae* infection. Hairy roots expressing GFP-PsAvh262 or GFP were inoculated with mycelia plugs of RFP-labelled wild-type *P. sojae* strain P6497 (P6497-RFP). Oospore production in the infected hair roots was observed under a confocal microscope (left panel), and lesion length was determined (middle panel) at 48 h.p.i. Expression of GFP or GFP-PsAvh262 was confirmed by western blotting using an anti-GFP antibody (right panel). (**d**) Expression of PsAvh262 in *N. benthamiana* enhanced infection of *Phytophthora capsici*. Leaf regions transiently expressing PsAvh262 or GFP, were inoculated with mycelia plugs of *P. capsici*. Infected leaves were stained with Trypan blue at 36 h.p.i. to visualize disease lesions (left panel) and the sizes of the lesions were determined (middle panel). Expression of GFP or PsAvh262-GFP was confirmed by western blotting using an anti-GFP antibody (right panel). Δ, non-specific band when using anti-GFP, which are common contaminants of western blots present in many published articles detecting GFP-fused proteins expressed in the plant cells^13^,^64^; #, PsAvh262 derived band; this may due to some unknown modification or degradation. Error bars represent the mean±s.d.(*n*=3) and asterisks (^**^or ^***^) denote significant differences (*P*<0.01 or *P*<0.001, assessed with one-way ANOVA). All experiments were repeated three times with similar results.

*PsAvh262* encodes a 123-amino-acid protein that has a secretion signal peptide and an RxLR motif^12^. PsAvh262 is conserved in various *P. sojae* strains (Supplementary Fig. 1a). To determine the possible role of PsAvh262 during *P. sojae* infection, we first analysed the expression patterns of *PsAvh262* at 1.5, 3, 6, 12 and 24 h post inoculation (h.p.i.) onto soybean hypocotyls. *PsAvh262* was highly expressed at early stages of infection with the maximal expression level observed at 1.5 h.p.i. (Fig. 1a).

We then investigated the contribution of PsAvh262 to *P. sojae* virulence through RNA silencing. Seven *PsAvh262*-silenced transformants of *P. sojae* strain P6497 (wild type) were obtained using polyethylene glycol (PEG)-mediated transformation. Quantitative RT–PCR (qRT–PCR) confirmed that the *PsAvh262* transcript levels in these transformants were significantly decreased, from 32 to 8% of the wild-type strain (Fig. 1b). All seven *PsAvh262*-silenced transformants showed reduced virulence on etiolated soybean seedlings, whereas two non-silenced transformants showed similar virulence as the wild-type strain (Fig. 1b). In addition, intense cell death symptoms were observed from soybean hypocotyls 9 h after inoculation with the *PsAvh262*-silenced transformants, whereas similar symptoms were not observed with the wild-type strain (Supplementary Fig. 1b). Silencing of *PsAvh262* did not change any developmental phenotypes of *P. sojae* (Supplementary Fig. 2). Taken together, these results indicate that PsAvh262 is an essential virulence effector that is required for *P. sojae* infection, possibly through its ability to suppress plant cell death.

To further examine the virulence function of PsAvh262, we transiently expressed it in soybean hairy roots and in agroinfiltrated *N. benthamiana* leaves, then inoculated the plant tissues with *Phytophthora*. Soybean hairy roots expressing PsAvh262-GFP or GFP were inoculated with mycelia of *P. sojae* strain *P6497RFP*, which constitutively expresses red fluorescent protein. As shown in Fig. 1c, the number of oospores formed on 500-μm-long hairy roots expressing *PsAvh262* was over three-fold more than on roots expressing GFP. Consistent with these results, transient expression of PsAvh262 in *N. benthamiana* leaves also increased their susceptibility to *P. capsici*; lesions on leaves transiently expressing PsAvh262 were significantly larger (*P*<0.01, one-way analysis of variance (ANOVA), *n*=5) than those on leaves expressing GFP (Fig. 1d).

In addition, PsAvh262 was able to suppress cell death in *N. benthamiana* triggered by a variety of elicitors, including the pro-apoptotic protein BAX, the *P. infestans* PAMP INF1 and the *P. sojae* RxLR effector Avh241 (Supplementary Fig. 1c). Collectively, these results establish that PsAvh262 can promote *Phytophthora* infection and can act as an inhibitor of programmed cell death.

### PsAvh262^60–82^ is essential for its virulence function

**Figure 2:**
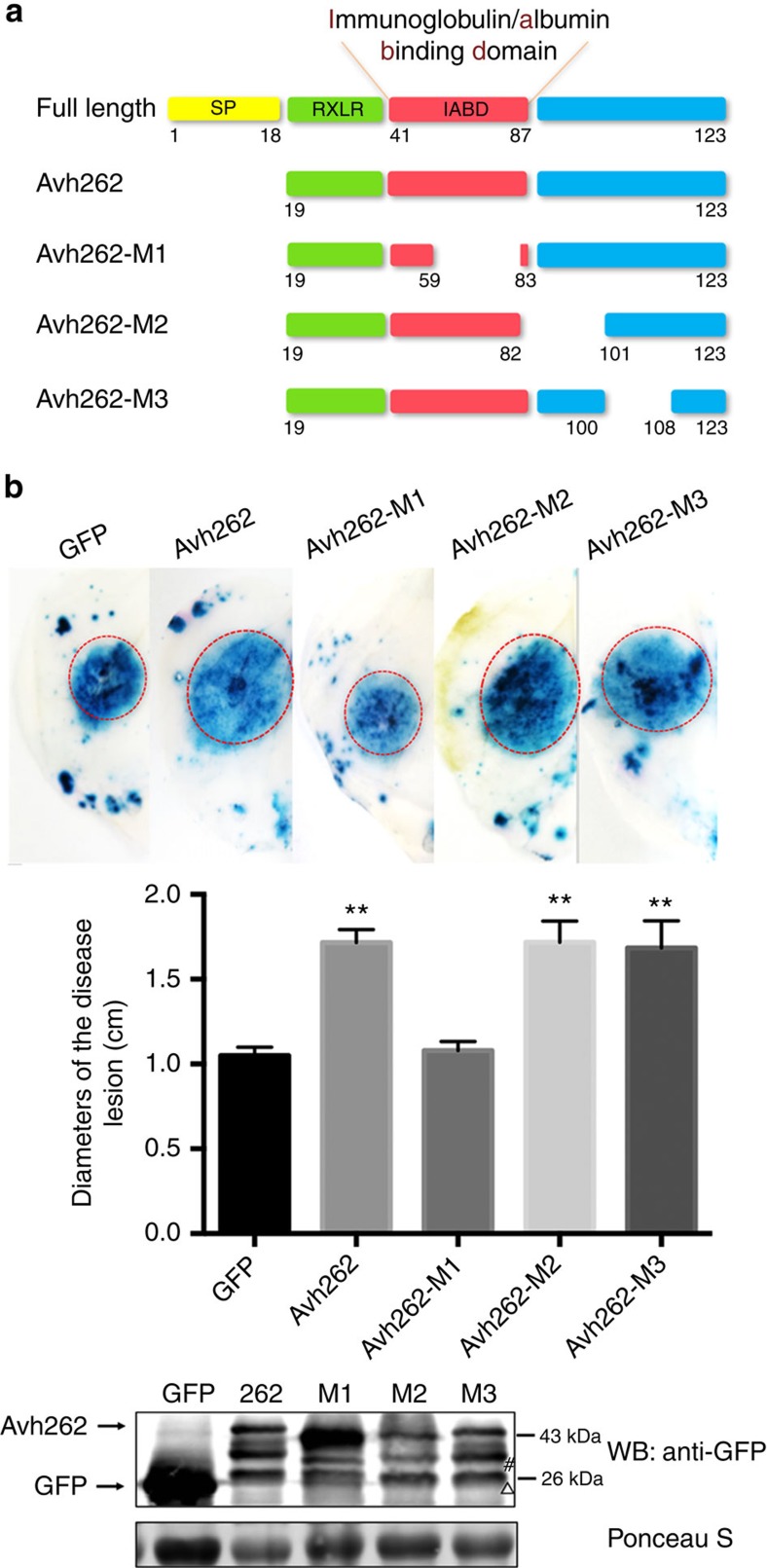
The potential immunoglobulin/albumin-binding domain is essential for the virulence function of PsAvh262. (**a**) Schematic diagram showing the protein structures of PsAvh262 and its deletion mutants. The putative immunoglobulin/albumin-binding domain (IABD) motif is deleted in PsAvh262-M1. SP, N-terminal secretion signal peptide. Numbers underneath each construct indicate amino acid positions. (**b**) PsAvh262-M1 lost the ability to promote *P. capsici* infection in *N. benthamiana*. Leaves transiently expressing GFP, PsAvh262 or PsAvh262 mutants were inoculated with *P. capsici*. The pictures were taken at 36 h.p.i. after disease lesions were stained with Trypan blue (upper panel). Sizes of the lesions were measured and subjected to statistical analysis (middle panel). Error bars represent the mean±s.d.(*n*=3), and asterisks (^**^) denote significant differences (*P*<0.01) between samples. The statistical significances of the pairwise differences between PsAvh262/mutants and the GFP control were assessed with one-way ANOVA. Similar phenotypes were observed in at least three independent experiments. Expression of GFP, GFP-PsAvh262 and the deletion mutants was confirmed by western blotting using an anti-GFP antibody. WB, western blot; M1, M2 and M3 represent PsAvh262-M1, PsAvh262-M2 and PsAvh262-M3, respectively. Δ, non-specific band when using anti-GFP; #, PsAvh262 derived band.

To identify functional domains in PsAvh262, we characterized potential motifs and its predicted secondary structure. A weak match to the immunoglobulin/albumin-binding domain (IPR009063; IABD) was identified within residues 41–87 using the Protein Homology/analog Y Recognition Engine V 2.0 (Phyre 2, http://www.sbg.bio.ic.ac.uk/phyre2/)^29^ (Supplementary Fig. 3a). This small three-helical domain has been found in various surface proteins in Gram-positive bacteria and shown to promote growth and virulence of *Peptostreptococcus magnus* (*P. magnus*)^30^. We examined three deletion mutants: PsAvh262-M1 (deletion of 60–82), which removed most of the possible IABD motif; PsAvh262-M2 (deletion of 83–100); and PsAvh262-M3 (deletion of 101–107) (Fig. 2a; Supplementary Fig. 3b). These mutants were transiently expressed in *N. benthamiana*, and then the plants were challenged with *P. capsici*. PsAvh262-M2 and PsAvh262-M3 functioned similarly to the wild-type PsAvh262 in promoting *Phytophthora* infection, whereas PsAvh262-M1 lost this virulence-promoting activity (Fig. 2b). These results suggest that the region 60–82 aa is essential for the virulence function of PsAvh262.

### PsAvh262 associates with plant ER-luminal BiPs

To identify the host targets of PsAvh262, N-terminal GFP-tagged PsAvh262 (without the N-terminal secretion signal peptide 1–18 aa) was transiently expressed in *N. benthamiana* leaves followed by immunoprecipitation (IP) using anti-GFP affinity beads. Immuno-purified proteins were then analysed by LC-MS/MS using the genome sequences of the closely related *Nicotiana tabacum*^13^. Among the *N. benthamiana* proteins that potentially associated with PsAvh262 (Supplementary Table 1), a protein similar to an ER-luminal BiP5 in *N. tabacum* (GenBank accession #729623) was selected for further analysis. Four BiPs, that is, NbBiP1, NbBiP2, NbBiP3 and NbBiP4, have been reported in *N. benthamiana*^31^. A search of the Sol Genomics Network (http://solgenomics.net/) using the NtBiP5 sequence as query identified an analogous protein (ID: NbS00040865g0006.1) in the *N. benthamiana* database with only one amino acid difference (a ‘V' versus an ‘I' at the position 13, 99.85% identity) (Supplementary Fig. 3). We therefore designated the PsAvh262-associated *N. benthamiana* protein as NbBiP5, which shares 76–96% identity in full-length amino-acid sequences with NbBiP1–NbBiP4, respectively (Supplementary Fig. 3). NbBiP5 also has homologues in soybean. The soybean *BiP* gene family consists of at least four members, *GmBiP1* (*Glyma08g02940*), *GmBiP2* (*Glyma08g02960*), *GmBiP3* (*Glyma05g36600*) and *GmBiP4* (*Glyma05g36620*), all of which are induced upon ER stress^32^. NbBiP5 shares 93.41, 91.77, 92.66 and 93.87% identity with GmBiP1–GmBiP4, respectively (Supplementary Fig. 4). Transcriptome analysis showed that *GmBiP1* has the highest transcription level, followed by *GmBiP4* (Supplementary Fig. 5). These two GmBiPs were utilized further in our study.

**Figure 3:**
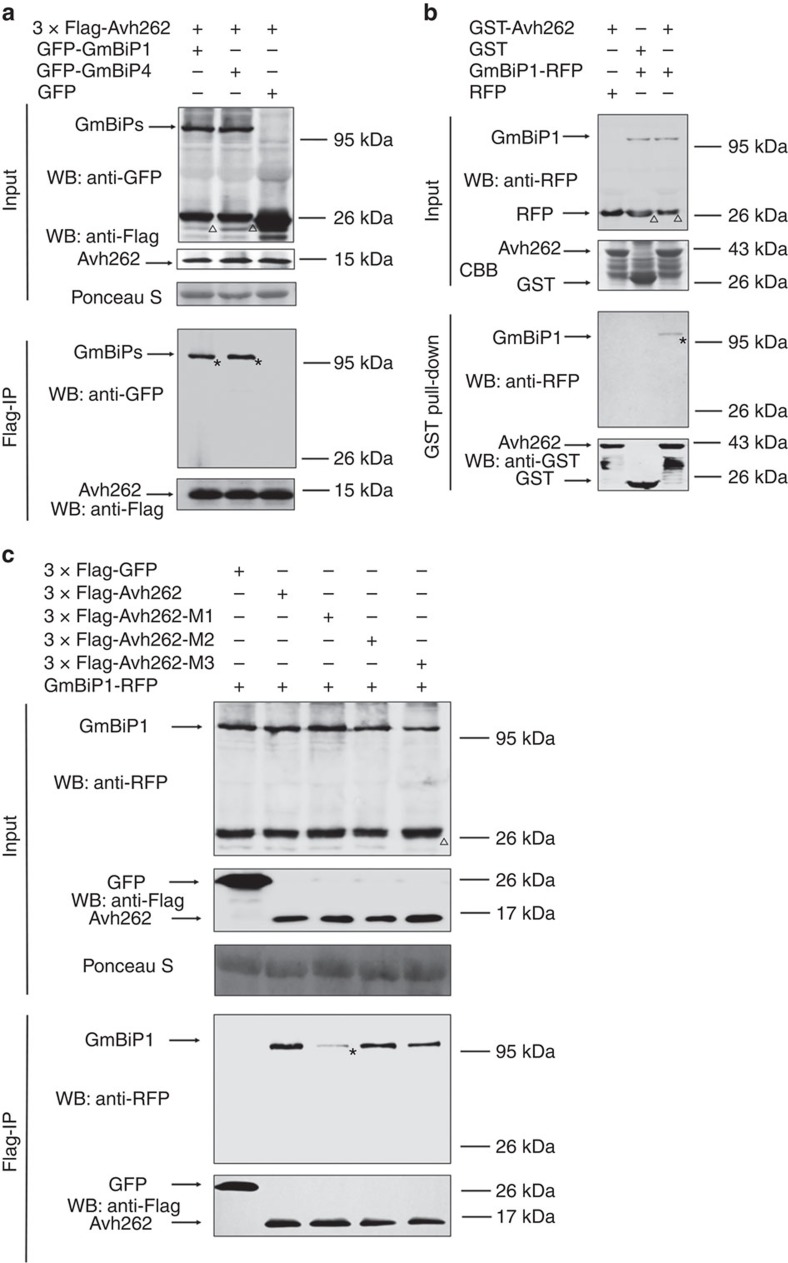
PsAvh262 interacts with plant endoplasmic reticulum luminal BiPs. (**a**) PsAvh262 interacts with soybean BiPs *in planta*. Total proteins were extracted from *N. benthamiana* leaves expressing 3 × Flag-PsAvh262 together with GFP-GmBiP1 or GFP-GmBiP4 (GFP was attached to the N terminus of the signal peptides of the GmBiPs). Protein complexes were pulled down using anti-Flag agarose beads and the co-precipitation of GFP-GmBiP1 or GFP-GmBiP4 was detected by western blotting using an anti-GFP antibody. (**b**) Proteins extracted from *N. benthamiana* leaves expressing GmBiP1-RFP (RFP was fused to the C-terminal HDEL sequence of GmBiP1) or RFP were incubated with *E. coli* homogenate containing GST-PsAvh262 or GST. Enrichment of GmBiP1-RFP in GST-PsAvh262-bound glutathione resins was detected using an anti-RFP antibody. (**c**) M1 deletion greatly reduces the interaction of PsAvh262 with GmBiP1. 3 × Flag-PsAvh262 or its mutants were co-expressed in *N. benthamiana* with GmBiP1-RFP. Protein complexes were pulled down using anti-Flag agarose gel, and co-precipitations of PsAvh262 and its mutants with GmBiP1 were detected using immunoblots. Membranes were stained with Ponceau S to confirm equal loading. *, the objective bands of GmBiPs. Δ, non-specific bands of GmBiPs when using anti-GFP or anti-RFP, similar to GFP-PsAvh262 protein. Similar results were obtained in at least three independent experiments.

To validate the association of PsAvh262 with NbBiP5 and GmBiPs, we carried out Co-IP assays (Fig. 3; Supplementary Fig. 6). GFP-PsAvh262 was co-expressed with NbBiP5-RFP or RFP in *N. benthamiana*. Total proteins were extracted from the infiltrated leaves and incubated with GFP-Trap_A beads (Chromotek, Germany). NbBiP5-RFP, but not RFP, was significantly enriched in the GFP-PsAvh262 precipitates (Supplementary Fig. 6a). Consistent with this result, GFP-GmBiP1, GFP-GmBiP2, GFP-GmBiP3 and GFP-GmBiP4, but not GFP, were also enriched in the 3 × Flag-PsAvh262 precipitates (Fig. 3a; Supplementary Fig. 6b) following transient expression in *N. benthamiana*. We next confirmed this interaction by a semi-*in vivo* GST-pull-down assay because we were unable to express *NbBiP5* or *GmBiP*s in *Escherichia coli* (*E. coli*). GmBiP1-RFP was expressed in *N. benthamiana* leaves and the total protein extracts were incubated with purified glutathione *S*-transferase (GST)-PsAvh262 expressed in *E. coli*. As showed in Fig. 3b, GmBiP1-RFP, but not RFP, was specifically enriched in GST-PsAvh262-bound glutathione beads. PsAvh262 also interacted with OsBiP3 from rice (Os02g02410) (Supplementary Fig. 6c). The interaction between PsAvh262-M1 and GmBiP1 was much weaker than the interaction with WT PsAvh262 (Fig. 3c), indicating that this region plays a key role in mediating the interaction between PsAvh262 and BiPs.

### PsAvh262 and GmBiP1 co-localize in the endoplasmic reticulum

**Figure 4:**
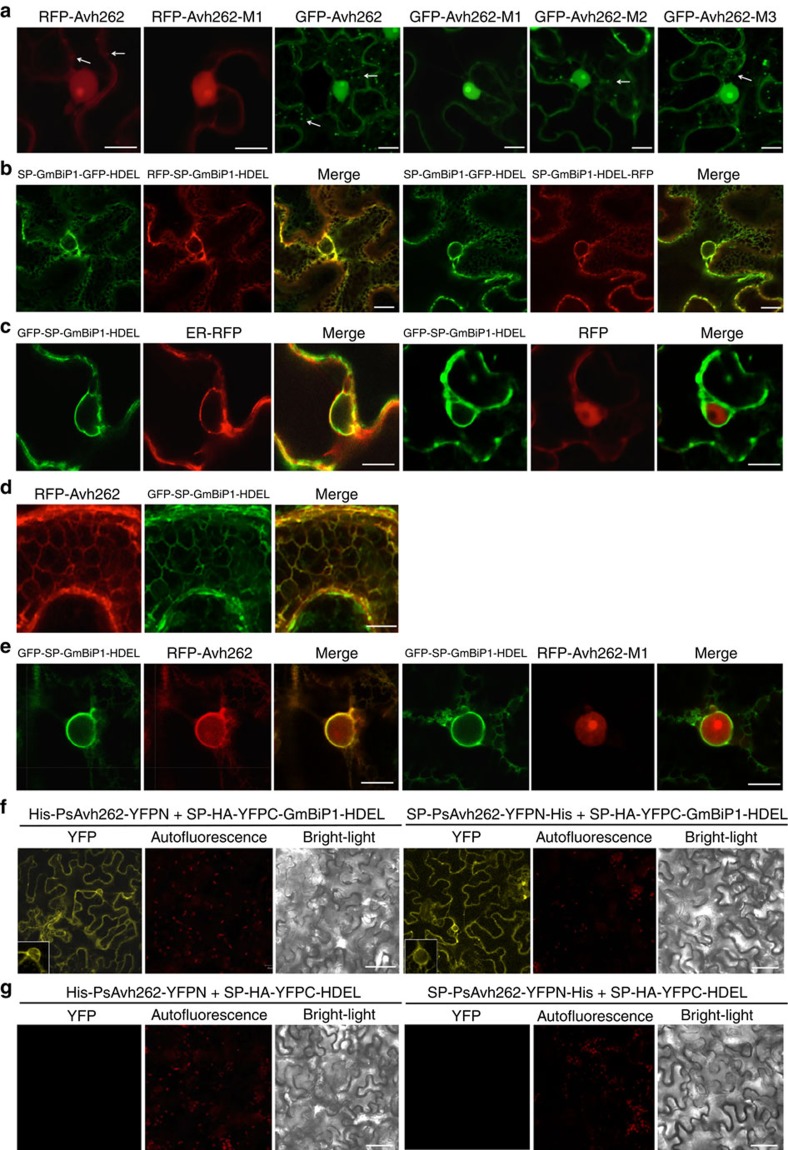
PsAvh262 and GmBiP1 co-localize to the endoplasmic reticulum. In all panels, proteins were expressed in *N. benthamiana* through agroinfiltration. Fluorescence was detected in epidermal cells of the infiltrated leaves by confocal microscopy at 48 h after agroinfiltration. Scale bars, 10 μm. (**a**) Localization of RFP- and GFP-fusions of PsAvh262 and mutants in the absence of GmBiP1. FPs were attached to the N terminus of PsAvh262 without its signal peptide. PsAvh262 and mutants M2 and M3 were primarily located in the nucleus and nucleolus, but also in punctate structures in the cell cortex. RFP-PsAvh262-M1 was no longer present in cell cortex. (**b**,**c**) Localization of GmBiP1 FP fusions to the ER. GmBiP1 fusions were targeted to ER-like structures irrespective of the placement of the FP (**b**). GFP-SP-GmBiP1-HDEL co-localizes with SP-RFP-HDEL, but not with RFP (**c**). (**d**,**e**) RFP-PsAvh262 and GFP-SP-GmBiP1-HDEL co-localize to the ER. Co-localization to the ER network (**d**) and to the peri-nuclear ER (**e**). The M1 deletion eliminates co-localization to the peri-nuclear ER. (**f**,**g**) Co-localization of PsAvh262 and GmBiP1 detected by bimolecular fluorescence complementation (BiFC) using the constructs shown in Supplementary Fig. 7a. Complementation is only observed with GmBiP1 constructs (**f**) but not control constructs (**g**).

To gain insights into the functional significance of the PsAvh262-GmBiP1 interaction, we determined the localization patterns of the proteins in plant cells. Wild-type or mutant PsAvh262 proteins (lacking the signal peptide) were co-expressed with GmBiP1 as a variety of GFP or RFP fusion proteins by agroinfiltration in *N. benthamiana*. Confocal microscopy showed that RFP-PsAvh262, GFP-PsAvh262 and their mutants (the FPs were attached to the N terminus of PsAvh262) were primarily localized in the nucleus when expressed in the absence of co-expressed BiPs (Fig. 4a). RFP-PsAvh262 and GFP-PsAvh262 also were present in punctate structures in the cell cortex^33^ (Fig. 4a). PsAvh262-M2 and PsAvh262-M3, but not PsAvh262-M1, also were present in punctate structures (Fig. 4a), indicating that the region deleted in M1 is required for the localization of PsAvh262 into the punctate structures. Consistent with the previously observed localization of BiPs to the ER^31^,^34^,^35^,^36^, we found that SP-GmBiP1-GFP-HDEL (that is, GFP inserted between BiP and its HDEL motif) localized in an ER-like network of subcellular structures including the perinuclear ER (Fig. 4b,c). Surprisingly, the localization of BiP was unchanged if the FP tag was placed onto the N terminus of the signal peptide or onto the C terminus of the HDEL motif (Fig. 4b) indicating that the localization of GmBiP1 is primarily controlled by sequences other than these two signals. Co-expression of GFP-SP-GmBiP1-HDEL with an ER marker (SP-RFP-HDEL) confirmed that the localization was to the ER (Fig. 4c).

Notably, when co-expressed with GFP-SP-GmBiP1-HDEL in *N. benthamiana*, RFP-PsAvh262 was co-localized with GFP-SP-GmBiP1-HDEL to the ER network (Fig. 4d) including the perinuclear ER (Fig. 4e). This re-localization in the presence of GmBiP1 was not observed for RFP-PsAvh262-M1 (Fig. 4e) or RFP (Fig. 4c). To further confirm the location of the GmBiP1-PsAvh262 interaction in plant cells, we conducted a bimolecular fluorescence complementation (BiFC) experiment. PsAvh262 (PsAvh262-YFPN, with or without its signal peptide) and GmBiP1 (SP-YFPC-GmBiP1-HDEL) were co-expressed in *N. benthamiana*. YFP fluorescence was observed exclusively from the ER (Fig. 4f), suggesting that PsAvh262 could reach and bind to BiP located inside the ER whether PsAvh262 was targeted to the cytoplasm or whether it was targeted to the ER by a signal peptide. No fluorescence complementation was observed when BiP was removed from the YFPC constructs (Fig. 4g). Taken together, our results suggest that the GmBiP1-PsAvh262 interaction occurs in the ER, though we cannot rule out that the interaction (also) occurs in the cytoplasm and that the complex subsequent can relocate to the ER.

### PsAvh262 and GmBiP1 co-localize around haustoria

**Figure 5:**
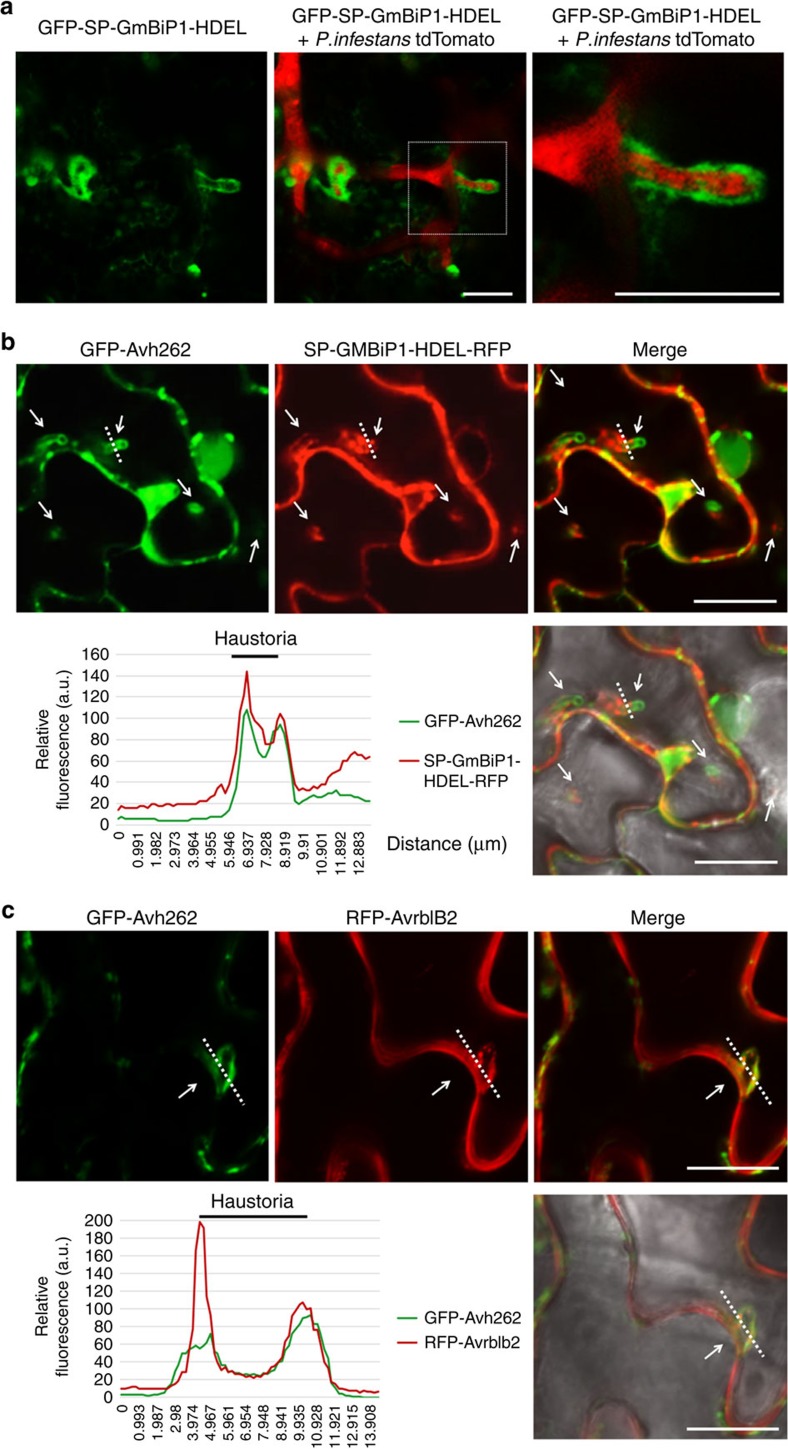
PsAvh262 and GmBiP1 co-localize around haustoria during infection. In all panels, proteins were expressed in *N. benthamiana* through agroinfiltration. The leaves were inoculated with *P. infestans* zoospores 2 days after agroinfiltration. Images were taken 2 days after inoculation with the relevant *P. infestans* strain. (**a**) GFP-SP-GmBiP1-HDEL accumulates around haustoria during infection by *P. infestans* strain 88069td expressing cytoplasmic tdTomato. Scale bars, 15 μm. (**b**) GFP-PsAvh262 and SP-GmBiP1-HDEL-RFP co-localized around haustoria following inoculation with untagged *P. infestans* strain T30-4. White arrows indicate the tips of haustoria. a.u., arbitrary units. Scale bars, 10 μm. (**c**) GFP-PsAvh262 and RFP-Avrblb2 (RFP attached to the N terminus of AvrBlb2 without its signal peptide) were co-localized around haustoria during infection by untagged *P. infestans* strain T30-4. In **b** and **c**, the fluorescence plots show the relative fluorescence along the dotted lines in the images. Arrowheads point to the tips of haustoria. a.u., arbitrary units. Scale bar, 10 μm.

To investigate the localization patterns of GmBiP1 and PsAvh262 during *Phytophthora* infection, we inoculated *N. benthamiana* leaves expressing GFP-SP-GmBiP1-HDEL with *P. infestans* strain 88069td tagged with cytoplasmic tdTomato. Confocal microscopy showed preferential accumulation of GFP-SP-GmBiP1-HDEL around haustoria in the infected leaves (Fig. 5a), suggesting that relocation of BiPs to the peri-haustorial region is associated with plant responses to infection. To determine PsAvh262 and BiPs were co-localized during infection, GFP-PsAvh262 (lacking its signal peptide) and SP-GmBiP1-HDEL-RFP were co-expressed in *N. benthamiana* leaves infected by untagged *P. infestans* strain T30-4. The results showed that both PsAvh262 and GmBiP1 localized to the perihaustorial region, though the distribution of GmBiP1 appeared biased towards the necks of the haustoria, while PsAvh262 appeared biased towards the tips (Fig. 5b). To determine if PsAvh262 was localized to the extra-haustorial membrane (EHM), we co-expressed GFP-PsAvh262 with the *P. infestans* RxLR effector Avrblb2 (RFP-Avrblb2), since Avrblb2 was reported to associate with the extra-haustorial membrane^13^,^37^. As shown in Fig. 5c, GFP-PsAvh262 localized around the haustoria coincident with Avrblb2, demonstrating that PsAvh262 was associated with the EHM. Interestingly, in contrast to Fig. 4a, PsAvh262 localized to the EHM in the absence of co-expression with GmBiP1 (Fig. 5c). Taken together, these results suggest that PsAvh262 and its target GmBiP1 co-localize around the haustoria and so may possibly influence infection.

### PsAvh262 stabilizes BiPs *in planta*

**Figure 6:**
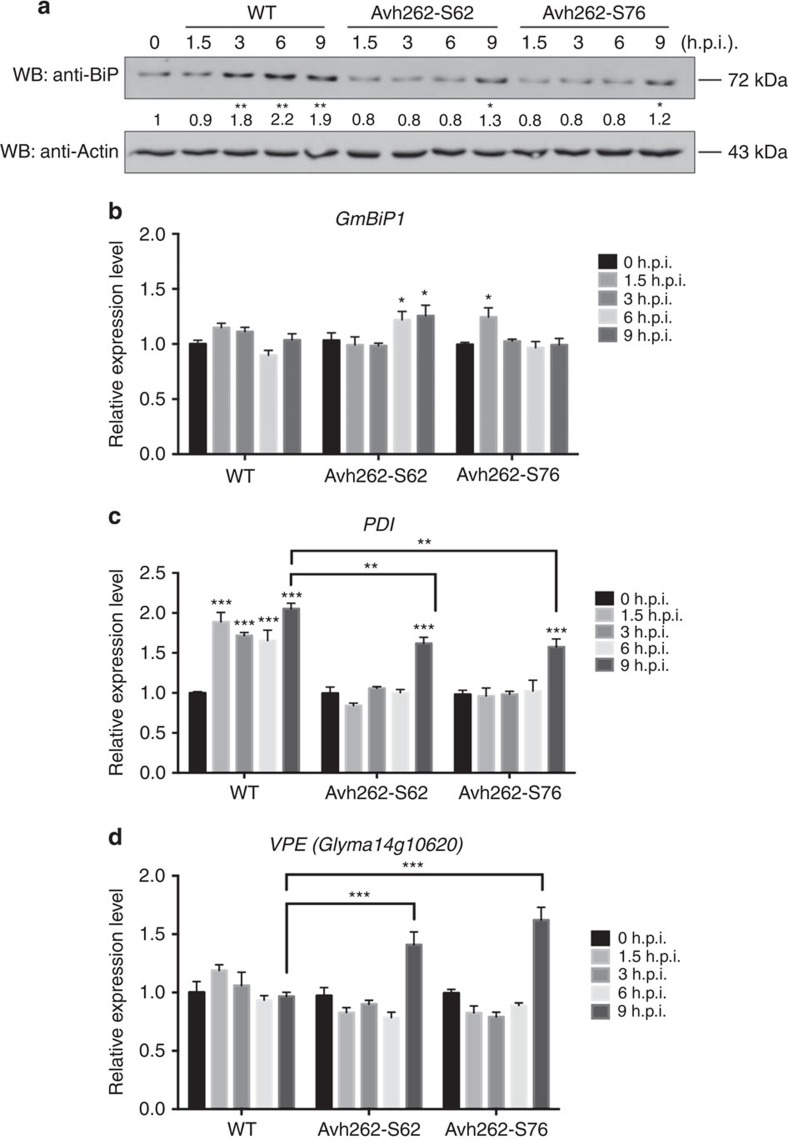
PsAvh262 attenuates endoplasmic reticulum stress-triggered cell death during *P. sojae* infection. (**a**) Accumulations of GmBiPs in soybean after hypocotyls were infected with *P. sojae* P6497 (wild-type) or two *PsAvh262*-silenced transformants (S62 and S76) were determined by western blotting using an anti-BiP antibody. Antibody against actin was used as an internal standard. Numbers below the blot indicate relative abundances of GmBiPs. Asterisks (* or **) denote significant differences (*P*<0.05 or *P*<0.01, one-way ANOVA, *n*=3) between samples. (**b**–**d**) Transcript abundances of ER-stress-related genes. The soybean housekeeping gene *CYP2* (TC224926) was used as an internal standard in each case. *GmBiP1* (**b**), *Protein disulfide isomerase* (*PDI*) (**c**) and *vacuolar processing enzyme* (*VPE*) (**d**) were determined by qRT–PCR in soybeans inoculated with wild-type or *PsAvh262*-silenced mutants of *P. sojae*. Error bars represent the mean±s.d.(*n*=3) and asterisks (*, ** or ***) denote significant differences (*P*<0.05, *P*<0.01 or *P*<0.001, respectively) between samples. The statistical significance of the pairwise differences between *PsAvh262*-silenced mutants and wild-type strain at 0 d.p.i. was assessed with one-way ANOVA. Similar results were observed in three independent experiments.

We next investigated whether the abundance of GmBiP1 could be affected by PsAvh262. For this purpose, we used western blots with anti-BiP antibodies to determine the endogenous levels of GmBiPs during *P. sojae* infection of soybean. The hypocotyls of etiolated soybean seedlings were inoculated with wild-type strain P6497 or with two independent *PsAvh262*-silenced transformants (Avh262-S62 and Avh262-S76). Total proteins were extracted from the infected tissues at 0, 1.5, 3, 6 and 9 h.p.i. We used 9 h.p.i. as our latest time point because *PsAvh262*-silenced transformants caused cell death at this time point. Abundances of GmBiPs increased from 3 to 9 h.p.i. in tissues inoculated with wild-type *P. sojae*, but much less in samples inoculated with the *PsAvh262*-silenced transformants (Fig. 6a). In addition, we found a small increase in GmBiP levels at 9 h.p.i. during infection by the *PsAvh262*-silenced transformants. Transcriptome analysis did not show significant differential accumulation of *GmBiP* transcripts during *P. sojae* infection (Supplementary Fig. 5). qRT–PCR results confirmed that the transcript levels of *GmBiP1* remained almost the same in soybean inoculated by either wild type or *PsAvh262*-silenced *P. sojae*, although there were slight fluctuations at some time points (Fig. 6b). These results suggest that PsAvh262 might have increased the stability of GmBiPs proteins during *P. sojae* infection, though we cannot rule out that stabilization of the GmBiPs is caused indirectly by the greater level infection by the wild-type strain, which results in a higher level of ER stress (see below).

**Figure 7:**
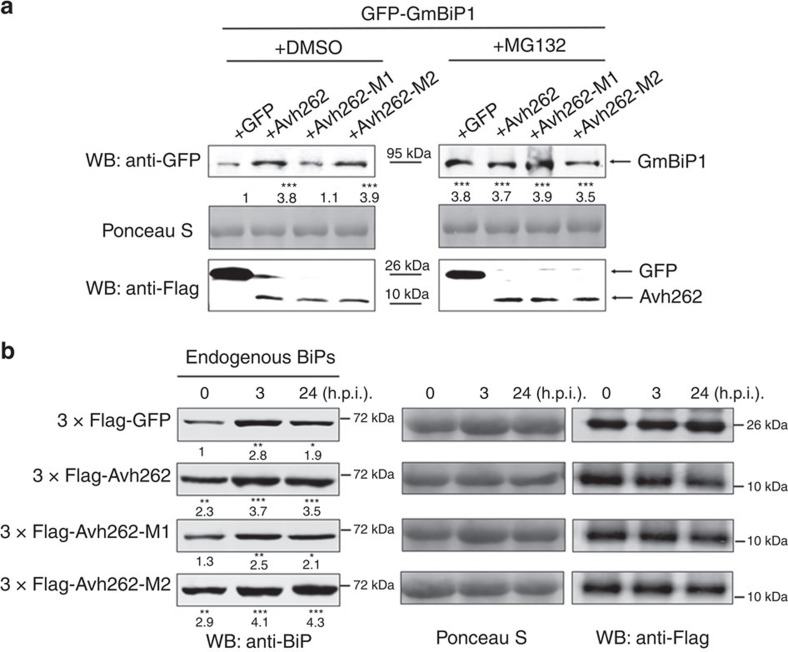
PsAvh262 stabilizes BiPs *in planta*. (**a**) Immunoblots showing the stabilization of GFP-GmBiP1 by PsAvh262 or the 26S proteasomal inhibitor MG132, but not by PsAvh262-M1. GFP-GmBiP1 (GFP was attached to the N terminus of the signal peptide of GmBiP1) was expressed in *N. benthamiana* leaves together with wild-type PsAvh262 or its mutants by agroinfiltration. MG132 (100 μM) or DMSO was infiltrated into the leaves at 24 h before protein extraction. Abundances of GFP-GmBiP1 were determined using an anti-GFP antibody. Numbers below the blot indicate relative abundances of GmBiP1. (**b**) Endogenous NbBiPs were stabilized by PsAvh262, but not by PsAvh262-M1 in *N. benthamiana* infected with *P. capsici*. *N. benthamiana* leaves expressing GFP, PsAvh262, PsAvh262-M1 or PsAvh262-M2 were inoculated with zoospore suspension of *P. capsici*, and the NbBiP levels were determined at 0, 3 and 24 h.p.i. Abundances of NbBiPs relative to GFP at 0 h were determined using an anti-BiP antibody. Numbers below the blots represent relative abundances of NbBiPs. Asterisks (*, ** or ***) denote significant differences (*P*<0.05, *P*<0.01 or *P*<0.001, respectively, one-way ANOVA, *n*=3) between samples. Similar results were observed in at least three independent experiments.

To more directly examine the effect of PsAvh262 on BiP stability, we compared the accumulation of NbBiP5-RFP in *N. benthamiana* when co-expressed with GFP-PsAvh262 or GFP using Agroinfiltration. As shown in Supplementary Fig. 8a, NbBiP5-RFP accumulated to a much higher level in the presence of GFP-PsAvh262 than when co-expressed with GFP. These results provide evidence that the presence of PsAvh262 can enhance the accumulation of NbBiP5. Plant ER-resident proteins can be efficiently transported out of the ER for degradation^38^ via the ubiquitin/26S proteasome pathway^39^. BiPs may also be regulated similarly by this mechanism. It is therefore possible that the interaction with PsAvh262 increases the retention of BiPs in the ER, thereby preventing their degradation. To test this hypothesis, we co-expressed NbBiP5 and GmBiP1 with wild-type PsAvh262 or the PsAvh262 mutants in *N. benthamiana*, and treated BiP-expressing leaves with the proteasomal inhibitor MG132. Increased accumulations of GmBiP1 (Fig. 7a) and NbBiP5 (Supplementary Fig. 8a) were observed in leaves treated with MG132 or co-expressing PsAvh262. Furthermore, MG132 treatment did not further increase the levels of GmBiP1 produced by Avh262 expression, suggesting that Avh262 and MG132 act via the same process (Fig. 7a). These results suggest that PsAvh262 prevents BiPs from being degraded *in planta* through the 26S proteasomal pathway. Interestingly, we found that PsAvh262-M1, but not PsAvh262-M2, lost the capacity to stabilize GmBiP1 (Fig. 7a; Supplementary Fig. 8b), indicating that direct PsAvh262-BiP interaction is required for BiP stabilization. PsAvh262-mediated inhibition of BiP degradation is specific because the *P. infestans* effector Avrblb2, which also associates with NbBiP5 (ref. 13), was unable to stabilize NbBiP5 (Supplementary Fig. 8b). Taken together, these results suggest that PsAvh262 stabilizes BiPs by preventing their degradation.

We next explored whether PsAvh262 influences the endogenous BiP levels *in planta* during *Phytophthora* infection. *N. benthamiana* leaves expressing GFP, PsAvh262, PsAvh262-M1 or PsAvh262-M2 were inoculated with zoospores of *P. capsici*, and the endogenous NbBiPs levels were determined at 0, 3 and 24 h.p.i. using anti-BiP antibodies. As shown in Fig. 7b, the accumulation of endogenous NbBiPs was enhanced during *P. capsici* infection; furthermore, NbBiP protein levels had an overall increase in leaves expressing PsAvh262 or PsAvh262-M2, but not PsAvh262-M1, at all three time points (Fig. 7b). These results support that PsAvh262 stabilizes BiPs during infection, and suggest that *P. capsici* may also produce effector(s) that play a similar role as PsAvh262.

### Avh262 suppresses ER stress-associated cell death

In a previous study, overexpression of GmBiP4 in soybean attenuated N-rich protein (NRP)-mediated cell death signalling by inhibiting the induction of several components in the pathway, such as homologues of the vacuolar processing enzyme (VPE)^40^. Protein disulfide isomerase (PDI), an ER-resident chaperone involved in mediating ERQC^40^, was shown to be induced at both the RNA and protein levels by ER stress^41^. Thus PDI can serve as a marker of ER stress. We therefore examined the transcript levels of *PDI* and *VPE* during *P. sojae* infection to monitor the induction of ER stress and the induction of ER stress-triggered cell death, respectively. qRT–PCR results showed that *PDI* transcript levels were upregulated nearly two-fold (*P*<0.01, one-way ANOVA, *n*=3) within 1.5 h after infection by wild-type *P. sojae* (Fig. 6c), whereas during infection by the *PsAvh262*-silenced transformants, minimal changes were observed in the levels of *PDI* transcripts prior to 9 h.p.i. PDI induction was observed at 9 h.p.i., but the levels were slightly lower than in tissues infected with the wild-type strain (Fig. 6c). These results suggest that the higher virulence of the wild-type strain results in earlier induction of ER stress than in the less virulent-silenced lines. The levels of *VPE* transcripts were not significantly changed during wild-type infection, however *VPE* levels were significantly upregulated at 9 h.p.i. during infection by the *PsAvh262*-silenced transformants (Fig. 6d), at which time cell death symptoms were observed. These results suggest that the *PsAvh262*-silenced transformants were no longer able to attenuate cell death resulting from the ER stress triggered by infection, even though the level of ER stress imposed by the silenced lines was less than that imposed by the wild type.

### BiPs regulate resistance and ER stress-triggered cell death

**Figure 8:**
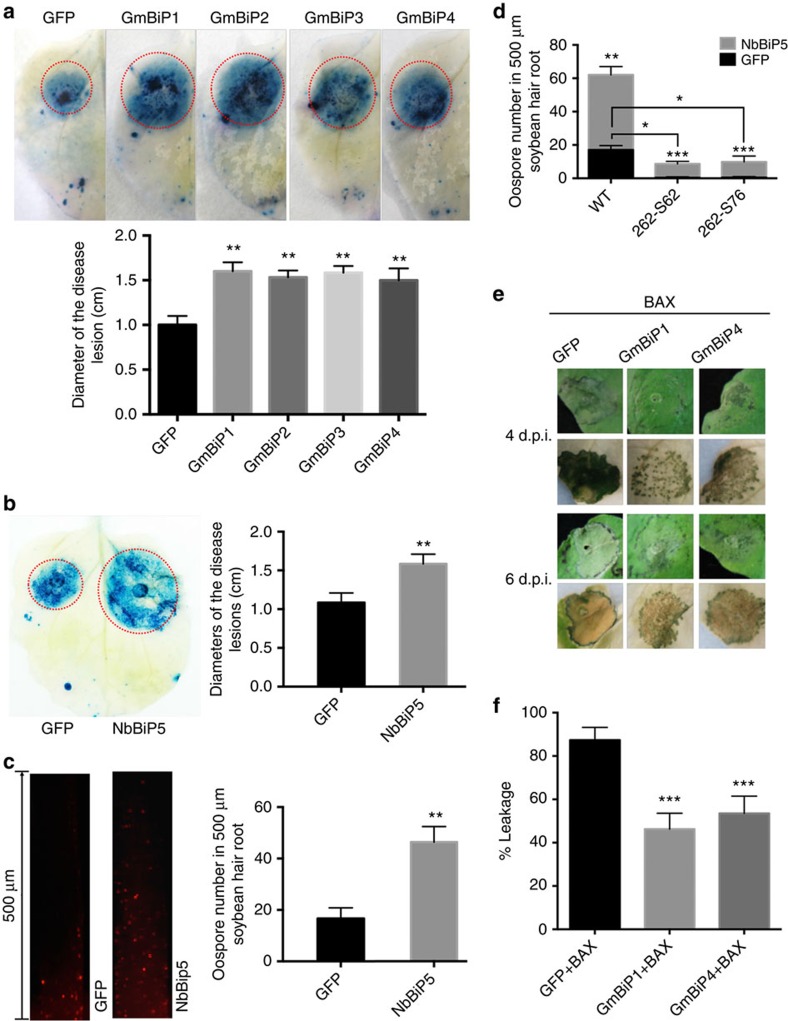
BiPs regulate plant resistance to *Phytophthora* and ER stress-triggered cell death in plants. (**a**) Expression of GmBiPs in *N. benthamiana* led to increased susceptibility to *P. capsici*. Leaves transiently expressing GFP-GmBiPs (GFP was attached to the N terminus of the signal peptide in each case) or GFP were inoculated with mycelia of *P. capsici*. Disease lesions were visualized by Trypan blue staining of the inoculated leaves at 36 h.p.i. (upper panel) and the sizes of the lesions were statistically analysed (lower panel). (**b**) Expression of GFP-NbBiP5 (GFP was attached to the N terminus of the signal peptide of NbBiP5) in *N. benthamiana* led to increased susceptibility to *P. capsici*. Disease lesions were visualized by Trypan blue staining at 36 h.p.i. (left panel) and the sizes of the lesions were statistically analysed (right panel). (**c**) Expression of GFP-SP-NbBiP5-HDEL in soybean hairy roots enhanced the infection by *P. sojae* strain P6497-RFP. Increased numbers of oospores developed in NbBiP5-expressing roots at 48 h after inoculation as shown by microscopic images (left panel) and statistical analysis (right panel). (**d**) Overexpression of GFP-SP-NbBiP5-HDEL can partly restore the virulence of *PsAvh262*-silenced *P. sojae* transformants in soybean roots. Soybean hairy roots expressing NbBiP5 or GFP were inoculated by wild-type *P. sojae* or *PsAvh262*-silenced transformants (262-S62, 262-S76), respectively. Increased number of oospores was developed in roots expressing NbBiP5 at 48 h after infection by *PsAvh262*-silenced *P. sojae* transformants. (**e**) Expression of GmBiP1 or GmBiP4 attenuated cell death triggered by BAX in *N. benthamiana*. GFP-SP-GmBiP1-HDEL, GFP-SP-GmBiP4-HDEL or GFP were expressed in *N. benthamiana* through agroinfiltration. Twelve hours later, the leaves were infiltrated again with *A. tumefaciens* cells carrying *HA* tagged *BAX*. Leaves were bleached with ethanol to visualize the cell death symptoms. Immunoblots confirmed the expression of GFP-SP-GmBiP1-HDEL and GFP-SP-GmBiP4-HDEL in *N. benthamiana* (lower panel). (**f**) Quantification of electrolyte leakage in *N. benthamiana* leaves expressing BAX together with GFP-SP-GmBiP1-HDEL, GFP-SP-GmBiP4-HDEL or GFP, 4 days after agroinfiltration. Electrolyte leakage from the infiltrated leaf discs was measured as a percentage of leakage from boiled discs. Error bars represent the mean±s.d. (*n*=3), and asterisks denote significant differences (*P<0.05; **P<0.01; ***P<0.001, one-way ANOVA). These experiments were repeated three times with similar results.

The influence of PsAvh262 on BiP levels during infection suggests that BiPs play a role in plant immunity. To test this, we transiently expressed GmBiPs and NbBiP5 (with GFP fused to the N terminus of the signal peptides) in *N. benthamiana* via agroinfiltration, and inoculated the leaves with *P. capsici* at 2 days post agroinfiltration. At 36 h.p.i., larger lesions were observed on leaves expressing each of the four GmBiPs (Fig. 8a) or NbBiP5 (Fig. 8b), indicating that increased accumulation of BiPs promoted *P. capsici* infection. Consistent with this result, overexpression of GFP-SP-NbBiP5-HDEL in soybean hairy roots also increased their susceptibility to *P. sojae* (Fig. 8c,d; Supplementary Fig. 9). In addition, the overexpression of GFP-SP-NbBiP5-HDEL could partly restore the susceptibility of the plants to *PsAvh262*-silenced *P. sojae* transformants. Taken together, our results suggest that PsAvh262-mediated virulence is affected through host BiPs (Fig. 8d; Supplementary Figs 9 and 10).

Silencing of *NbBiPs* individually in *N. benthamiana* is difficult due to their highly conserved nucleotide sequences (Supplementary Fig. 11). It has been shown that silencing of *NbBiPs* caused developmental defects, including curling of leaves and the appearance of many pits on leaves^42^. However, the lower leaves in *NbBiPs*-silenced plants could still be used for pathogen inoculation as the developmental defects were only observed in the newly developing plant parts^42^. We silenced all *NbBiPs* by PVX-based virus-induced gene silencing (VIGS) to investigate their roles in plant defence during *Phytophthora* infection. The mRNA levels of *NbBiPs* were reduced by 80–90% in several independent VIGS plants (Supplementary Fig. 12a). The *NbBiPs*-silenced plants showed a cell death phenotype as previously described^43^ (Supplementary Fig. 12b). When infected with *P. capsici*, the sizes of disease lesions on *NbBiPs*-silenced leaves were significantly smaller than those on the non-silenced leaves (Supplementary Fig. 12c). Consistent with the smaller lesion size, a greatly reduced biomass of *P. capsici* was observed in *NbBiPs*-silenced leaves (Supplementary Fig. 12d). Taken together, these results suggest that BiPs are negative regulators of plant resistance to *Phytophthora* infection, though we cannot rule out that the physiological changes in the BiP-silenced plants indirectly resulted in resistance.

Since PsAvh262 suppresses BAX-triggered cell death and interacts with all four soybean BiPs (Fig. 3 and Supplementary Figs 1c; 6), we also examined the influence of NbBiP5, GmBiP1 and GmBiP4 on BAX-triggered cell death. ER stress is a component of the mechanism by which BAX triggers cell death^44^. Our results showed that these three proteins (expressed with GFP attached to the N terminus of the signal peptide) all significantly alleviated BAX-triggered cell death and electrolyte leakage compared with a GFP control when expressed in *N. benthamiana* (Fig. 8e,f; Supplementary Fig. 13). Thus, these results suggest that BiPs can negatively regulate cell death triggered by ER stress.

## Discussion

**Figure 9:**
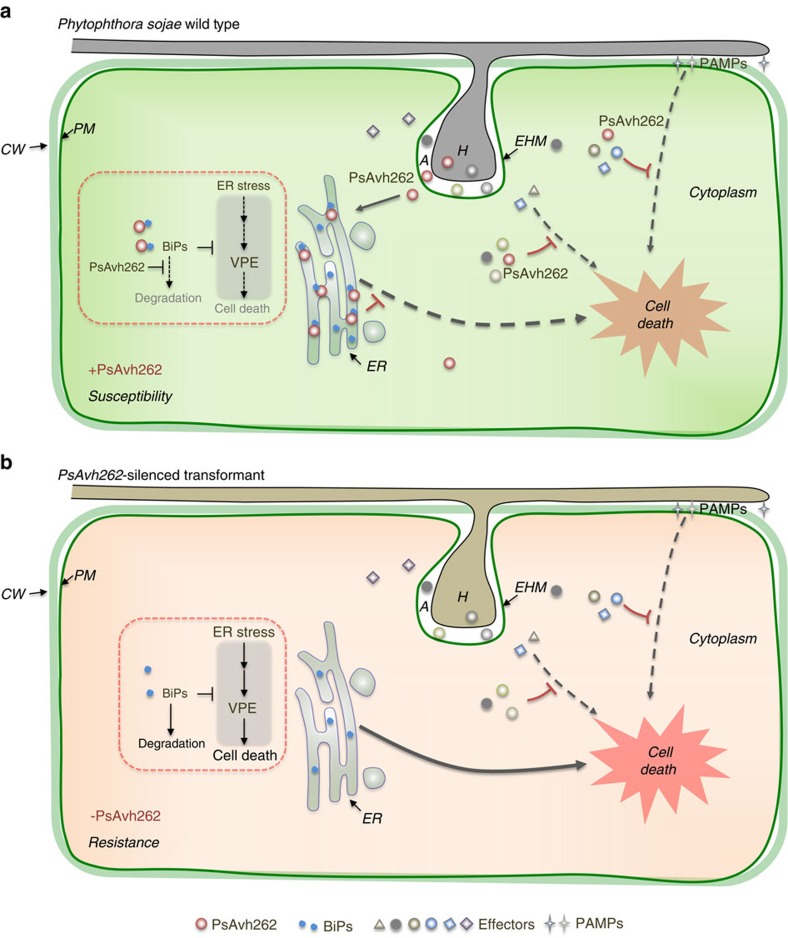
A schematic summary illustrating PsAvh262 suppression of ER stress-mediated immunity by stabilization of plant BiPs during infection. During infection, *P. sojae* produces haustoria, through which effectors are secreted and translocated into host cells. In infected ‘haustoriated' cells, the host plasma membrane remains intact but is invaginated by the invading hyphae and becomes the extrahaustorial membrane (EHM). *Phytophthora* infection can activate the ER stress response in the host plants, which subsequently triggers cell death to block infection. (**a**) In the initial biotrophic phase, PsAvh262 associates and stabilizes host BiPs in the ER to attenuate the ER stress-triggered cell death. As such, PsAvh262 acts as an essential virulence factor to enhance *P. sojae* colonization and infection. Avh262 (along with many other RxLR effectors) can also suppress PAMP- and effector-triggered cell death, but it is unknown if this activity of Avh262 is mediated by the BiP interaction. (**b**) In the absence of PsAvh262, *P. sojae* is no longer able to attenuate the ER stress-induced cell death, which prevents infection. A, apoplast; CW, cell wall; EHM, extrahaustorial membrane; ER, endoplasmic reticulum; H, haustoria; PM, plasma membrane.

Hemibiotrophic pathogens initially establish a biotrophic relationship with their hosts, but at later stages of the infection they kill the host cells. During the initial biotrophic phase, the pathogen feeds on viable host tissues for nutrition via haustoria^10^,^45^,^46^; thus, efficient mechanisms must be employed to suppress or evade host defenses at this stage, especially programmed cell death (PCD)^47^. To achieve this, *Phytophthora* pathogens deliver a large repertoire of effectors into host cells to suppress immune responses including PCD. PCD is a widespread mechanism for plants to cope with environmental stresses such as attacks by pathogens. Identifying the target host factors of these effectors and their functions will lead to detailed understanding of how infection is established. Using a combination of genetic, cellular and biochemical approaches, we established the *P. sojae* RxLR effector PsAvh262 as a critical virulence factor for infection in soybean. One function of PsAvh262 is to suppress cell death triggered by PAMPs and effectors. We identified BiPs from soybean as one of the major virulence targets of PsAvh262. PsAvh262 could also interact with *N. benthamiana* and rice homologues of GmBiPs. Our experiments showed that PsAvh262 stabilizes host BiPs from degradation by an MG132-sensitive mechanism, that is probably the ERAD pathway. Furthermore, overexpression of BiPs could suppress PCD triggered by BAX; BAX relies partly on ER-stress to trigger PCD. Finally, our results showed that PsAvh262-silenced *P. sojae* lines were ineffective in suppressing PCD associated with infection-induced ER stress. Even when the level of ER stress was reduced by the poor virulence of the *PsAvh262*-silenced *P. sojae* lines (as measured by PDI transcript levels; Fig. 6c), the silenced lines were ineffective in suppressing PCD (as measured by VPE transcript levels; Fig. 6d). Together, these results suggest that stabilization of BiPs by PsAvh262 acts to attenuate PCD triggered by infection-associated ER stress (Fig. 9).

There is growing evidence that different forms of PCD may harm or benefit pathogens depending on the timing and nature of the cell death mechanism, and whether the pathogen has physiologically adapted to benefit from the dying tissue^26^. Among the various cellular stress responses that induce plant PCD, the ER stress response governs resistance to diverse environmental stresses^43^,^48^. BiPs play both broad and specific roles in regulating ER stress-triggered cell death^41^,^49^. For example, they act as moderators of PCD and master regulators of the UPR during ER stress^27^,^42^,^50^,^51^,^52^. Thus, attenuation of ER stress-triggered PCD by PsAvh262 through its interaction with BiPs represents a novel and effective counter-defence strategy by *P. sojae*. BiP-targeting is likely of broad significance, given that BiPs function in systemic acquired resistance (SAR)^27^, pathogen recognition receptor (PRR) biosynthesis^31^,^53^ and regulation of cell surface receptor-mediated innate immunity^54^. Silencing of *BiPs* in *N. benthamiana* resulted in delays in PCD induced by *Xanthomonas oryzae* pv. *oryzae*, but not by *Pseudomonas syringae* pv. *tomato* strain DC3000 (ref. 42). Silencing of ERD2a and/or ERD2b reduced accumulation of BiPs in the ER, resulting in increased sensitivity of plants to ER stress and exacerbation of PCD induced by non-host pathogens^42^. In contrast, Carvalho *et al*.^41^ found that soybean transgenic lines overexpressing GmBiP4 displayed enhanced or accelerated PCD induced by *Pst* DC3000^41^. In *Arabidopsis*, transgenic plants overexpressing another ER chaperone, calreticulin 2 (CRT2), were more susceptible to *Pst* DC3000^55^. Thus, the expression level of ER chaperones may increase or decrease plant susceptibility, depending on the pathogen involved. Our results, combined with the observations of these authors, highlight the ER stress response as a key battleground in the struggle between plants and pathogens to control plant cell death.

We found that PsAvh262 co-localizes with BiPs to the ER in uninfected plant cells, and also to the EHM during infection of *N. benthamiana* by *P. infestans* (Fig. 5). Co-localization of BiPs and PsAvh262 to the ER displayed some unexpected features. First, the localization of GmBiP1 to the ER was unaffected by fusion of GFP or RFP to the N terminus of its signal peptide, or to the C terminus of its HDEL ER retention signal (Fig. 4b,c). This suggests that the BiPs may reach the ER by a post-translational pathway^56^. Second, our BiFC results revealed that PsAvh262 could co-localize with BiP even when BiP was targeted directly to the ER by an unobstructed signal peptide (Fig. 4f,g). This observation suggests that even with an unobstructed signal peptide, BiP passes through the cytoplasm where it may interact with PsAvh262. In the absence of co-expressed GmBiP1, PsAvh262 appeared to display little localization to the ER (Fig. 4a). A small amount of localization to punctate structures was observed; this might represent localization to the ER mediated by endogenous BiPs, however the identity of the punctate structures was not confirmed in our study. Furthermore, the PsAvh262 M1 deletion mutant that does not interact with BiP did not colocalize to the ER (Fig. 4e). These results suggest that cytoplasmic PsAvh262 is dependent on BiP binding to localize to the ER. When PsAvh262 was targeted directly to the ER by the signal peptide of GmBiP1, it could also complex with GmBiP1 as judged by our BiFC results (Fig. 4f,g). Thus PsAvh262 may have the ability to find and bind to BiP whether it is in the cytoplasm or the ER.

The most commonly accepted idea about RxLR effector entry is that it involves endocytosis from the apoplast or extra-haustorial matrix into host cells^2^,^3^. If PsAvh262 enters via endocytosis and then is trafficked from the endosomes into the ER, then it would be in the appropriate compartment for interaction with ER-localized BiPs. On the other hand, if PsAvh262 was delivered directly into the host cell cytoplasm, either from endosomes or across the plasma membrane, then it could interact with BiPs in the cytoplasm and from there be targeted to the ER, as shown in Fig. 4. The targeting of PsAvh262 (and BiPs) to the EHM surrounding the pathogen during infection (Fig. 5) may also be relevant to its delivery pathway. PsAvh262 did not require co-expression with BiP to be targeted to the EHM. Thus PsAvh262 might be delivered directly to the EHM during infection, and might rendezvous with BiP at that location prior to delivery to the ER.

Several other oomycete RxLR effectors have been reported to localize to the ER in host cells including nine RxLR effectors from *Hyaloperonospora arabidopsidis* (*Hpa*)^57^ and the *P. infestans* RxLR effector Pi03192. In particular, Pi03192 interacts with NAC transcription factors in the ER and prevents re-localization of NAC transcription factors from the ER to the Nucleus^58^. It is unknown how those effectors reach the ER.

PsAvh262 may have additional functions than the binding of BiPs. A substantial fraction of PsAvh262 accumulated in plant nuclei and nucleoli. Furthermore, additional candidate targets were identified in the immuno-purification experiments that revealed the BiPs as targets (Supplementary Table 1). In a broader context, it is notable that many Gram-positive bacteria express surface proteins that bind host immunoglobulins or serum proteins^59^. As an example of this important function, *Peptostreptococcus magnus* expresses an albumin-binding surface protein that binds human serum albumin to promote bacterial growth and virulence^30^. Given that plants do not have immunoglobulins or albumins, our finding that PsAvh262 has a weak match to an IABD domain within the region corresponding to mutant M1 is of interest. It would be of interest to explore effectors containing potential IABD and investigate whether they target yet-to-be-discovered plant proteins to promote infection.

## Methods

### Plasmid construction

The *PsAvh262* gene was cloned using complementary DNA from *P. sojae*. Full-length *BiP* genes were cloned from *N. benthamiana*, soybean cultivar Williams 82 and rice cDNAs using gene-specific primers containing at least 15 bp of a vector sequence available on each side of the cloning site (Supplementary Table 2). The amplified fragments were ligated into pBINGFP2 (a plasmid containing green fluorescent protein, GFP), pCAM1300-Flag and pCAM1300-RFP with the In-Fusion HD Cloning Kit (Clontech, Mountain View, CA, USA). *PsAvh262* without a signal peptide and the *PsAvh262* deletion mutants were amplified using combinations of primers (Supplementary Table 2). The amplicons were prepared using the appropriate restriction enzymes (Supplementary Table 2) and ligated into pBINGFP2, pCAM1300-Flag, pCAM1300-RFP, pEGX4T-2, PVX (pGR107) and pTOR. The RFP insert was ligated into *Sal*I- and *Kpn*I-digested pCAM1300 to yield pCAM1300-RFP. The fragments used to generate *PsAvh262* deletion mutants 3 × Flag-Avh262, 3 × Flag-Avh262-M1, 3 × Flag-Avh262-M2 and 3 × Flag-Avh262-M3 were synthesized by Nanjing Genscript (Nanjing, China) and ligated into pBINGFP2 and pCAM1300 with the In-Fusion HD Cloning Kit. Individual colonies for each construct were tested for inserts by PCR, and selected clones were verified by sequencing.

### *A. tumefaciens* infiltration assays and plant growth

Constructs were introduced into *Agrobacterium tumefaciens* (*A. tumefaciens*) strain GV3101 by electroporation. The transformed bacterial cells were grown on LB agar plates supplemented with appropriate antibiotics. Individual colonies were verified by PCR using both vector- and gene-specific primers. For agroinfiltration assays, recombinant *A. tumefaciens* strains were grown at 28 °C in a shaking incubator at 200 r.p.m. After 36–48 h, bacterial cells were spun down by centrifugation, and resuspended in MES buffer (10 mM MgCl_2_ and 1 mM MES, pH 5.6). The resuspended *A. tumefaciens* cells were diluted and mixed with P19 silencing suppressor^60^ in a 1:1 or 1:1:1 ratio (depending on the number of constructs) to a final OD_600_=0.3 for each construct. For stabilization assays, MG132 or DMSO (a control) was mixed or individually infiltrated into plant leaves after the agroinfiltration for 24 h. *N. benthamiana* plants were grown in a greenhouse for 4–6 weeks at 25 or 20 °C under a 16 h/8 h day/night photoperiod. For VIGS, the two largest leaves of four-leaf-stage *N. benthamiana* plants were pressure-infiltrated with *A. tumefaciens* strain GV3101 containing either the VIGS construct PVX-NbBiPs or the PVX-GFP control with a concentration of OD_600_=1.0. Three weeks after agroinfiltration, plants were used for infection assays or for analysis of gene silencing efficiency using qRT–PCR.

### Immunopurification of transiently expressed proteins

Leaves of 4–6-week-old *N. benthamiana* plants were agroinfiltrated with pBINGFP2-PsAvh262 (and pBINGFP2 as the control) and the P19 silencing suppressor in a 1:1 ratio at a final OD_600_=0.3 for each construct. Two days after agroinfiltration, the leaves were frozen in liquid nitrogen and ground to a fine powder using a mortar and pestle. Proteins were extracted using lysis buffer (10 mM Tris-Cl (pH 7.5), 150 mM NaCl, 0.5 mM ethylenediaminetetraacetic acid (EDTA), 0.5% NP-40) plus 1 mM phenylmethylsulfonyl fluoride and a protease inhibitor cocktail (Sigma-Aldrich, St Louis, MO, USA) by mixing 1 g leaf tissue with 2 ml of lysis buffer. The samples were centrifuged at 4 °C for 15 min at 14,000*g* and the supernatant was transferred to a new tube. For mass spectrometry analysis of proteins co-purified with the PsAvh262 proteins, 5 ml of total protein extract was incubated at 4 °C for 3 h with 30 μl of GFP-Trap_A beads (Chromotek, ABIN509397, Planegg-Martinsried, Germany). For the other immunopurification experiments, 2 ml of total protein extract was incubated at 4 °C for 2 h with 20 μl of GFP-Trap_A beads. The beads were then collected by centrifugation at 2,500*g* and washed five times in 1 ml of washing buffer (10 mM Tris-Cl (pH 7.5), 150 mM NaCl and 0.5 mM EDTA). Bound proteins were eluted by adding 50 μl of 0.2 M glycine (pH=2.5) for 30 s with constant mixing, followed by centrifugation. The supernatant was transferred to a fresh tube, and 5 μl of 1 M Tris base (pH=10.4) was added for neutralization. To increase elution efficiency, this step was repeated. The resuspended beads were boiled for 10 min at 95 °C to dissociate the immunocomplexes from the beads. The beads were then collected by centrifugation at 2,500*g* for 2 min at 4 °C and SDS–PAGE (SDS–polyacrylamide gel electrophoresis) was performed with the supernatant.

### GST pull-down

PsAvh262 was inserted into the vector pGEX4T-2 (GE Healthcare Life Science, Little Chalfont, Buckinghamshire, UK) and expressed in *E. coli* strain BL21. Soluble total proteins (GST-Avh262) were incubated with 50 μl glutathione-agarose beads (GE Healthcare Life Science) at 4 °C for 2 h, supernatant was removed and the beads were washed three times with ice-cold phosphate-buffered saline (PBS) buffer. Next, GmBiP1-RFP protein extracted from GmBiP1-RFP-expressing *N. benthamiana* plants was added to the above beads for the incubation at 4 °C for 2 h. After six times of washing with 1 × PBST, the SDS sample loading buffer was added to beads and heated to 100 °C for 5 min for the immunoblot analysis.

### Western blotting

Proteins from the sample lysate were fractionated by SDS–PAGE. The separated proteins were transferred from the gel to an Immobilon-PSQ polyvinylidene difluoride membrane (pretreated with methanol for 15 s; Millipore) using a transfer buffer (20 mM Tris, 150 mM glycine). The membrane was then blocked using PBS(pH 7.4) containing 3% non-fat dry milk for 30 min at room temperature with 50 r.p.m. shaking, followed by one wash with PBST (PBS with 0.1% Tween 20). Anti-Flag (1:2,000; #F3165; Sigma-Aldrich) and anti-BiP (1:1,000; #sc-33757; Santa Cruz Biotechnology), anti-GFP (1:2,000; #M20004; Abmart), anti-RFP (1:1,000; #5f8; Chromotek), anti-HA (1:2,000; #M20003; Abmart), anti-myc (1:2,000; #M20002; Abmart) and anti-actin (1:2,000; #M20009; Abmart) antibodies were added to PBSTM (PBS with 0.1% Tween 20 and 3% non-fat dry milk) and incubated at room temperature for 90 min, followed by three washes (5 min each) with PBST. The membrane was then incubated with a goat anti-mouse IRDye 800CW antibody (Odyssey, no. 926-32210; Li-Cor) at a ratio of 1:10,000 in PBSTM at room temperature for 30 min with 50 r.p.m. shaking. The membrane was washed three times (5 min each) with PBST, once for 5 min with PBS, and then visualized by excitation at 780 and 800 nm. Full-size images are presented in Supplementary Fig. 14.

### *P. sojae* transformation and characterization

*P. sojae* strain P6497 (race 2) was routinely grown and maintained on V8 agar. Stable transformation was performed as the following protocol^12^. Two or three-day-old mycelia, cultured in pea broth medium, were washed in 0.8 M mannitol, then placed in enzyme solution (0.4 M mannitol, 20 mM KCl, 20 mM MES, pH 5.7, 10 mM CaCl_2_, 10 mg ml^−1^ β-1.3 glucanase and 5 mg ml^−1^ cellulysin, and incubated for 35–45 min at 25 °C with ∼100 r.p.m. shaking. The protoplasts were harvested by centrifugation at 1,500 r.p.m. for 3–5 min and resuspended in W5 solution (5 mM KCl, 125 mM CaCl_2_, 154 mM NaCl and 31 mg ml^−1^ glucose) at a concentration of 2 × 10^6^ protoplasts per ml or higher. After 30 min, the protoplasts were centrifuged at 1,200*g* for 5 min and resuspended in an equal volume of MMg solution (0.4 M mannitol, 15 mM MgCl_2_ and 4 mM MES, pH 5.7) to allow protoplasts to swell. To each of 1 ml MMg solution, 25 μg transforming DNA was added and incubated for 10 min on ice. Then, three aliquots of 580 ml each of freshly made polyethylene glycol solution (40% (v/v) polyethylene glycol 4,000, 0.3 M mannitol and 0.15 M CaCl_2_) were slowly pipetted into the protoplast suspension and gently mixed. After 20 min incubation on ice, 10 ml pea broth containing 0.5 M mannitol was added, and the protoplasts were incubated overnight to regenerate. The regenerated protoplasts were suspended in liquid pea agar containing 0.5 M mannitol and 25 μg ml^−1^ G418 and plated. The visible colonies could be observed after 2–3 days incubation at 25 °C. All transformants were propagated on V8 agar with 50 μg ml^−1^ G418 at 25 °C.

For DNA or RNA extraction, mycelia of *P. sojae* transformants were cultured in V8 liquid, harvested and ground into a powder in liquid nitrogen. Genomic DNA was isolated from mycelia using the Multisource Genomic DNA miniprep kit (Axygen, Corning, NY, USA) following the manufacturer's protocol. To screen *PsAvh262*-silenced transformants, total RNA was extracted from the mycelia and used to generate cDNA. The resultant cDNA was used as template in the quantitative real-time PCR assays to determine the expression levels of *PsAvh262*.

The virulence of *P. sojae* transformants was determined by inoculation of etiolated soybean seedlings (Chinese susceptible cv. HF47). Stationary mycelial cultures were grown in liquid V8 broth in 90 mm Petri dishes at 25 °C in the dark for 2 days. The hyphae were repeatedly washed with sterile distilled water and then incubated in the dark at 25 °C for 4 to 8 h until sporangia developed on most of the hyphae and zoospores were released. Soybean seedlings were grown in the dark for 4 days at 25 °C (16 h per day) and 16 °C (8 h per day). Hypocotyls of etiolated soybean seedlings (9–12 per assay) were inoculated 2–3 cm from the base of the cotyledon with 200 zoospores and then incubated in the dark at 25 °C. Tissue samples from five seedlings were pooled and used to quantify oospores.

### Analysis of transcription profiles

Transcription data of the four *GmBiP* genes were obtained from RNA-seq for infected soybean roots (cultivar: Williams 82) by *P. sojae* (P6497) zoospores (NCBI Sequence Read Archive (SRA) accession: SRP073278). Except 0 h.p.i., using only soybean roots, the others (0.5, 3, 6 and 12 h.p.i.) were mixed samples of soybean roots and *P. sojae*. RNA was extracted using the EZNA total RNA kit I (Omega Bio-Tek, USA) and used to make RNA-seq libraries with the Illumina TruSeq RNA Sample Preparation Kit v2 following the manufacturer's recommendations. These libraries were sequenced using Illumina HiSeq 2000 in paired-end mode with a read length of 90 bp. The sequence reads were mapped to the soybean Williams 82 genome (Phytozome database, https://phytozome.jgi.doe.gov/; Glycine max Wm82.a2.v1), using TopHat 2.1.0 (http://ccb.jhu.edu/software/tophat/index.shtml). The gene expression levels were calculated using Cufflinks 2.2.1 (http://cole-trapnell-lab.github.io/cufflinks).

### SYBR green qRT–PCR

The RNA was quantified using a spectrophotometer (ND-1000; NanoDrop, Wilmington, DE, USA). To remove contaminating genomic DNA in the RNA samples, 3 μg of total RNA was treated with two units of RNase-free DNase I (Takara Bio Inc., Otsu, Japan) at 37 °C for 30 min. First-strand cDNA was synthesized using Moloney murine leukemia virus reverse transcriptase (RNase-free) and an oligo (dT) 18 primer (Invitrogen, Carlsbad, CA, USA). qPCR was performed in 20 μl reactions containing 20 ng of cDNA, 0.2 mM gene-specific primer or the reference actin gene, 10 μl of SYBR Premix ExTaq (TaKaRa) and 6.8 μl of deionized water. PCR was performed on an ABI Prism 7500 Fast Real-Time PCR System (Applied Biosystems Inc., Foster City, CA, USA) under the following conditions: 95 °C for 30 s and 40 cycles at 95 °C for 5 s and 60 °C for 34 s, followed by a dissociation step, that is, 95 °C for 15 s, 60 °C for 1 min and 95 °C for 15 s.

### *Phytophthora* infection assays in *N. benthamiana*

*N. benthamiana* leaves were collected 36 or 48 h after agroinfiltration and maintained on half-strength Murashige and Skoog (MS) medium in a Petri dish. Infiltrated regions were inoculated with 2.5% V8 juice agar plugs (diameter=0.5 cm) infected with fresh *P. capsici* LT263 mycelia as previously described^61^. For assays of *P. capsici* virulence in *NbBiP5*-silenced and non-silenced leaves, zoospores were used as inoculum and prepared as follows. Mycelia of *P. capsici* were grown on 20% V8 juice agar in 9-cm disposable Petri dishes for 2 days at 25 °C. The mycelia were rinsed twice with sterile distilled water, flooded with sterile distilled water to cover the mycelia, and then kept overnight at 25 °C to release zoospores. Zoospore concentration was determined by counting the number of zoospores under a microscope. *N. benthamiana* leaves were inoculated with 1,500 zoospores and incubated in a sealed Petri dish for 36 h at 25 °C in the dark. After that, the inoculated leaves were stained by Trypan blue as previously described^62^. Stained leaves were photographed and the diameters of the lesions were photographed and measured. Total DNA was extracted from *P. capsici*-infected regions (diameter=4 cm) was isolated at 36 h.p.i. and used for real-time PCR to quantify the ratio of host-to-pathogen biomass, using primers specific for *N. benthamiana* and *P. capsici* actin genes (Supplementary Table 2). Three independent biological replicates were included. The *P. infestans* strain 88069td or T30-4 was inoculated in the leave of *N. benthamiana* for the microscopical observation as previously described^13^.

### Transformation of soybean and *P. sojae* infection assays

Soybean cotyledons were inoculated with *A. rhizogenes* K599 carrying pBINGFP2, pBINGFP2-PsAvh262, or pBINGFP2-NbBiP5 (ref. 63). Individual cotyledons were collected from 10-day-old soybean seedlings. Detached cotyledons were surface-sterilized with 70% ethanol before a small roughly circular (diameter=∼0.4 cm) cut was made in each cotyledon ∼0.3 cm from the petiole end. The wounded cotyledons were then transferred to a sterile Petri plate containing 0.8% agar. *A. rhizogenes* cells grown in LB medium supplemented with kanamycin were washed and resuspended in 10 mM MgCl_2_ to a final concentration of OD_600_=0.3. Twenty microlitres of the cell suspension was directly spotted onto the wound site of each cotyledon. The inoculated cotyledons were incubated in a growth chamber at 22 °C under high humidity with a 16 h/8 h light/dark regime. Hairy roots usually started to generate from the wound sites at ∼3 weeks post inoculation. GFP-tagged PsAvh262 and NbBiP5 or GFP were expressed under the control of the CaMV 35S promoter. Fluorescence microscopy was used to select GFP-, GFP-PsAvh262 or GFP-NbBiP5 expressing roots. Green fluorescence was detected in hairy roots using a fluorescence stereomicroscope (Leica MZ FLIII, Wetzlar, Germany) with a GFP2 filter (excitation 480/40 nm, emission 510 nm). Expression of GFP-PsAvh262, or GFP-NbBiP5 proteins in hair roots was confirmed by western blotting. Four-week-old hairy roots were infected by *P. sojae-RFP* mycelia, and the number of oospores was determined by microscopy at 48 h.p.i.

### Electrolyte leakage assay

Cell death was assayed by measuring ion leakage from leaf discs^61^. For each sample, five leaf discs (1 cm diameter) were floated on 5 ml distilled water for 3 h at room temperature. Then the conductivity of the bathing solution was measured with a conductivity metre (Con 700; Consort, Tutnhout, Belgium) to give ‘value *A*'. The leaf discs were then returned to the bathing solution and boiled in sealed tubes for 25 min. After cooling the solution to room temperature, the conductivity was measured again to obtain ‘value *B*'. For each measurement, ion leakage was expressed as per cent leakage, that is (value *A*/value *B* × 100). All assays were repeated three times.

### Confocal microscopy

Patches of agroinfiltrated *N. benthamiana* leaves were cut and mounted in water and analysed using an LSM 710 laser scanning microscope with a × 20, × 40 or × 60 objective lens (Carl Zeiss, Jena, Germany). GFP or RFP fluorescence was observed at excitations of 488 or 561 nm, respectively. Bright field in soybean epidermal cells in Supplementary Fig. 1b was detected using a × 20 objective lens (IX71; Olympus, Tokyo, Japan).

### Protein structure analysis

Protein secondary structures and protein surface accessibility were predicted using NetSurfP 1.1 (http://www.cbs.dtu.dk/services/NetSurfP). The protein secondary structure class (alpha-helix, beta-strand and coil) predicted to have the greatest probability was considered as the result. Surface accessibility results were obtained directly from the default output. The putative IABD domain was identified using Phyre2 (http://www.sbg.bio.ic.ac.uk/phyre2)^29^.

### Data availability

The sequences data (PsAvh262, GmBiPs and NbBiP5) that support the finding of this study have been deposited in GenBank (http://www.ncbi.nlm.nih.gov/genbank/), Phytozome (https://phytozome.jgi.doe.gov/pz/portal.html) and Sol Genomics Network (http://solgenomics.net/), respectively. The primary accession codes are JN254253.1 (PsAvh262, GenBank); Glyma08g02940 (GmBiP1), Glyma08g02960 (GmBiP2), Glyma05g36600 (GmBiP3) and Glyma05g36620 (GmBiP4) in Phytozome; NbS00040865g0006.1 (NbBiP5, Sol Genomics Network). RNA-seq data described in this study has been deposited in the NCBI sequence read archive under accession code SRP073278. The authors declare that all other data supporting the findings of this study are available from the corresponding author on request.

## Additional information

**How to cite this article:** Jing, M. *et al*. A *Phytophthora sojae* effector suppresses endoplasmic reticulum stress-mediated immunity by stabilizing plant Binding immunoglobulin Proteins. *Nat. Commun.* 7:11685 doi: 10.1038/ncomms11685 (2016).

## Supplementary Material

Supplementary Information Supplementary Figures 1-14 and Supplementary Tables 1-2
