# Supplementary material for: A Phytophthora sojae effector suppresses endoplasmic reticulum stress-mediated immunity by stabilizing plant Binding immunoglobulin Proteins
Source: Nat Commun. 2016 Jun 3;7:11685. doi: 10.1038/ncomms11685 (PMC4895818; doi:10.1038/ncomms11685)
Supplement: Supplementary Information — Supplementary Figures 1-14 and Supplementary Tables 1-2 [file ncomms11685-s1.pdf]

Supplementary Fig. 1

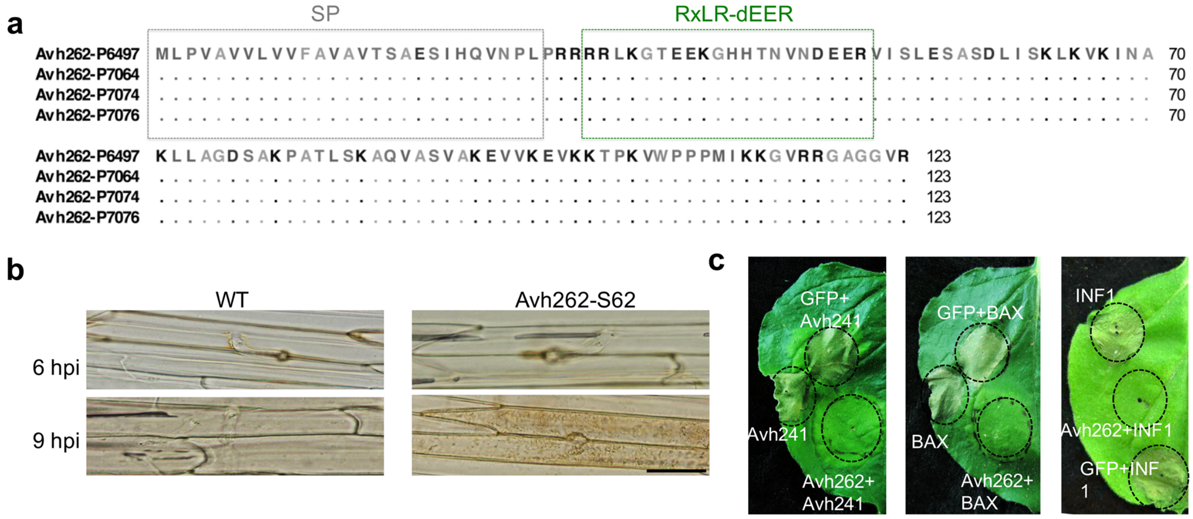

Supplementary Fig. 1. PsAvh262 is a conserved effector in *Phytophthora sojae* strains that can suppresses plant cell death. Related to Fig. 1.

(a) PsAvh262 sequences were identified in four *P. sojae* strains (P6497, P7064, P7074 and P7076). PsAvh262 encodes a 123-amino-acid protein with a predicted signal peptide (SP) and an RxLR-dEER motif. (b) Cell death produced by infection of hypocotyls with *P. sojae* strain Avh262-S62 silenced for *PsAvh262*. Avh262-S62 infected successfully host cells at 6 hours post-inoculation (hpi), but induced cell death at 9 hpi. The wild type strain (P6497) could invade host cells without triggering cell death at 9 hpi. Bar = 50 μm. (c) PsAvh262 suppresses cell death triggered by BAX, INF1, and Avh241 in an agroinfiltration assay in *N. benthamiana*. Cells carrying PsAvh262 were infiltrated 24 hours before cells carrying the challenge constructs. Similar phenotypes were observed in at least three independent experiments.

Supplementary Fig. 2

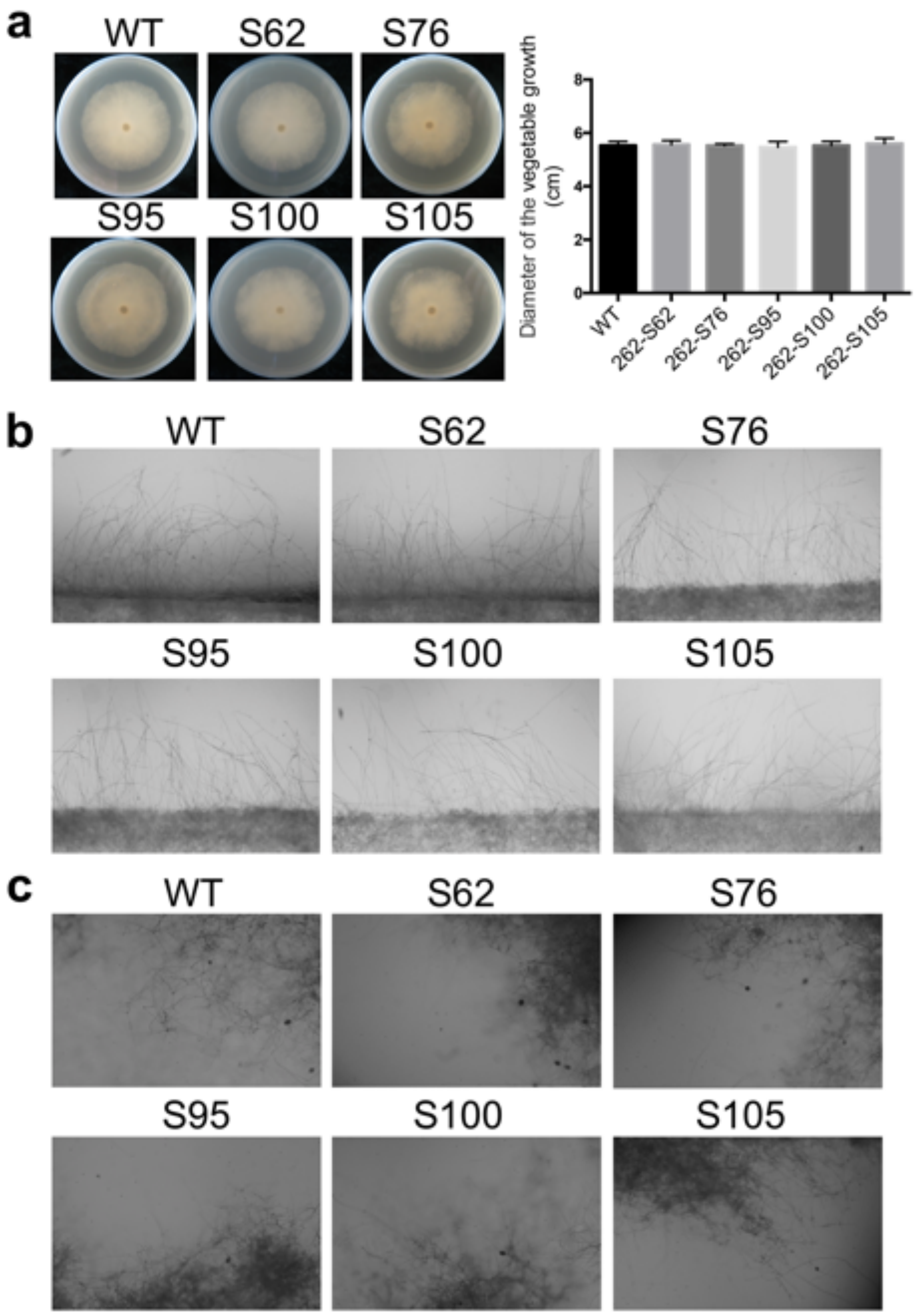

Supplementary Fig. 2. Silencing of *PsAvh262* does not affect mycelial growth or zoosporangium formation.

(a) Mycelial growth was not altered in the *PsAvh262*-silenced transformants. Wild-type and *PsAvh262*-silenced transformants were incubated on 10 % V8 medium at 25°C for 6 days and then pictures were taken. Colony growth rate was calculated based on the radius of WT and transformants colonies. Error bars represent the mean ± s.d.(n=3). The statistical significances were assessed with one-way ANOVA. The experiments were repeated at least three times independently. (b) The hyphal morphology was not altered in the *PsAvh262*-silenced transformants, as judged by light microscopy. (c) Zoosporangium formation was normal in the *PsAvh262*-silenced transformants.

Supplementary Fig. 3

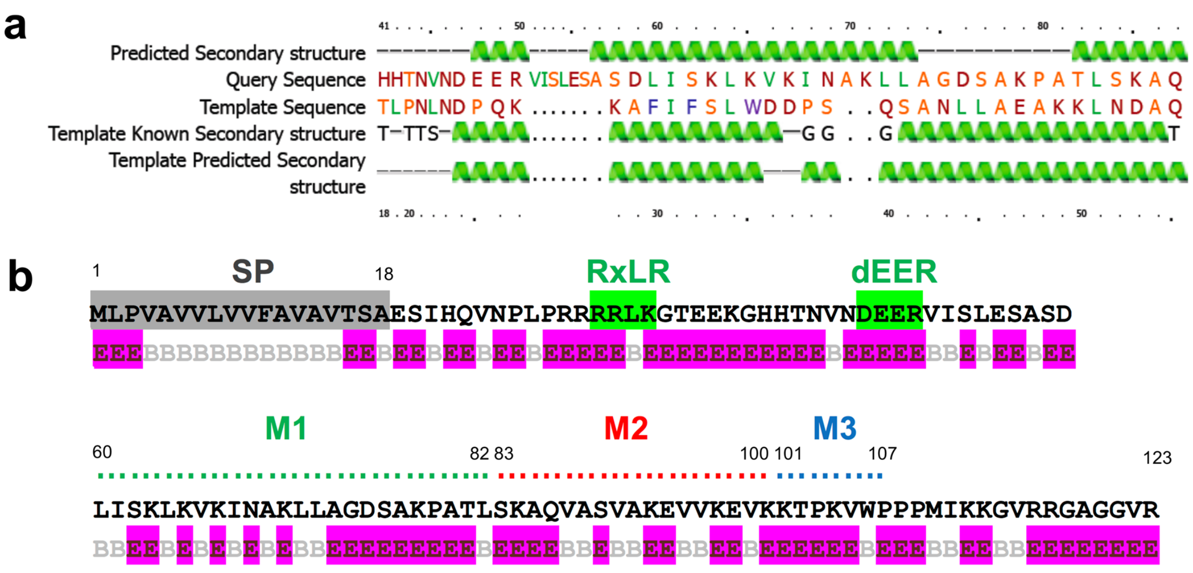

Supplementary Fig. 3. PsAvh262 contains a predicted immunoglobulin/albumin-binding domain.

(a) Identification and secondary structure of the predicted immunoglobulin/albumin-binding domain in PsAvh262, obtained using Phyre2[28]. The query sequence is PsAvh262 and the template sequence is d1lp1a (B domain of IgG-binding protein Z of *Staphylococcus aureus*). The confidence of the match was 40.2%. (b) Surface accessibility of PsAvh262. SP, RxLR, and dEER are motifs, M1, M2, M3 indicate deletion mutants. B, buried; E, exposed.

Supplementary Fig. 4

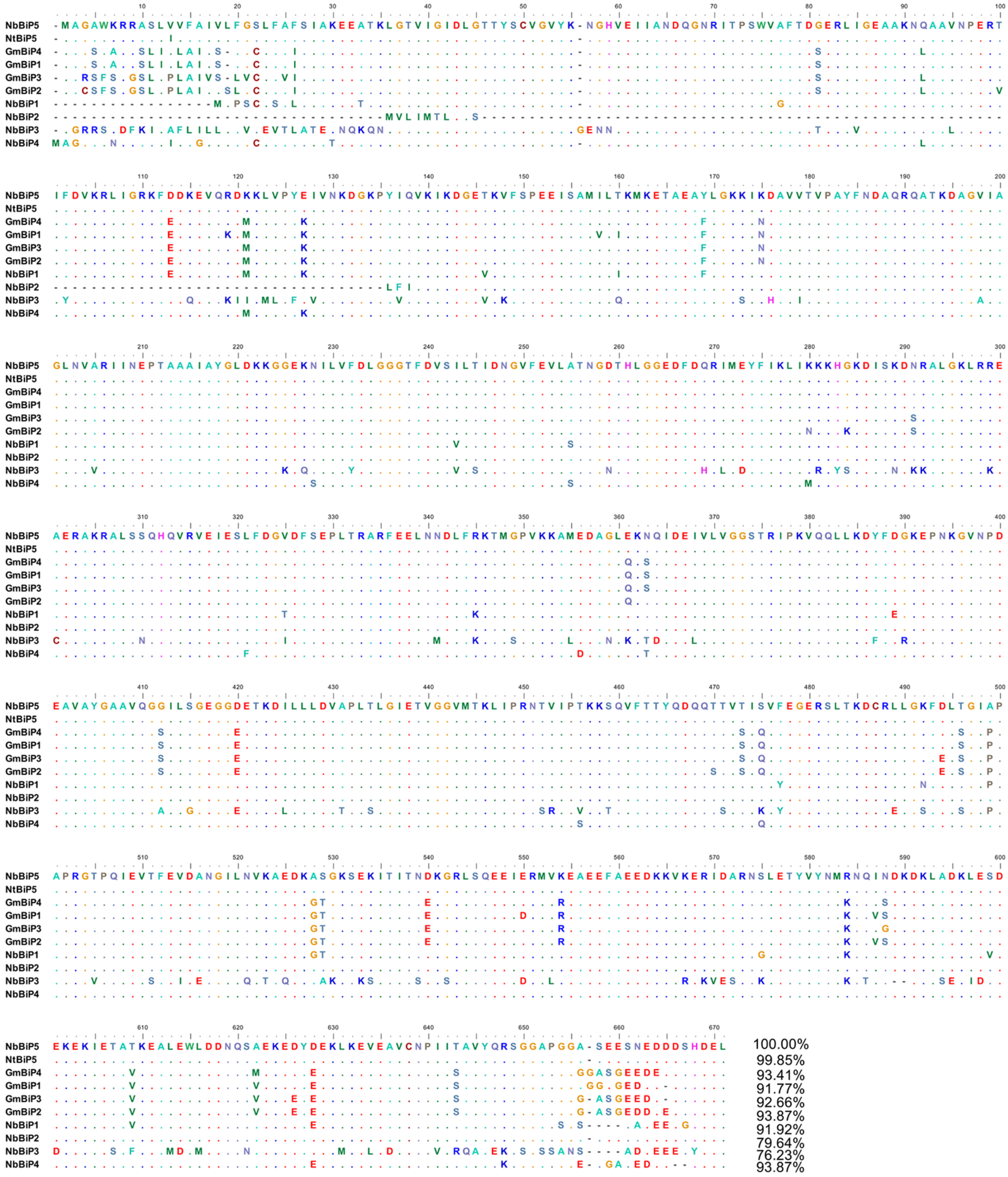

Supplementary Fig. 4. Alignment of amino acid sequences of NtBiP5, NbBiPs and GmBiPs.

The multiple sequence alignment was generated with ClustalW (http://www.ch.embnet.org/software/ClustalW.html). The amino acid identities with NbBiP5 were shown.

Supplementary Fig. 5

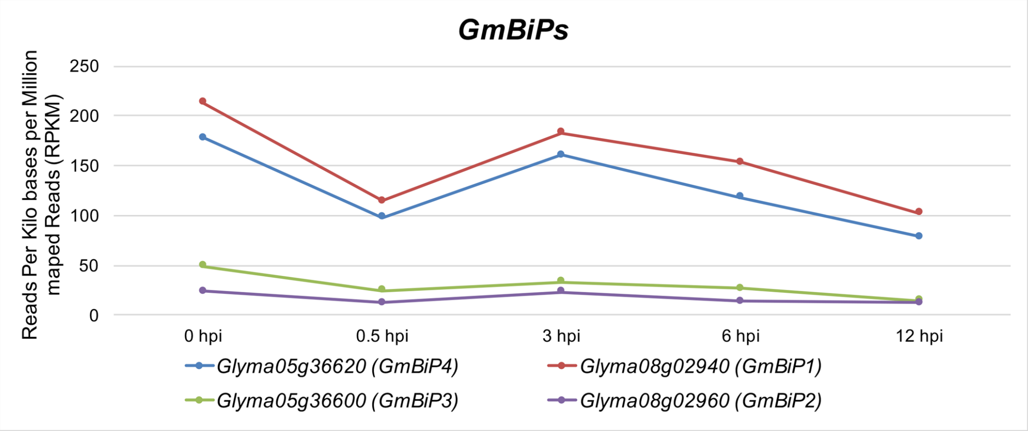

Supplementary Fig. 5. Transcription profiles of *GmBiPs* during the early stage of *P. sojae* infection.

Transcription levels of *GmBiPs* were determined by an RNA-seq analysis (NCBI Sequence Read Archive (SRA) accession: SRP073278). The hypocotyl of susceptible soybean cultivar Williams 82 was used in this work, infected by *P. sojae* strain P6497. Transcriptome analysis showed that GmBiP1 and GmBiP4 had the highest expression levels.

Supplementary Fig. 6

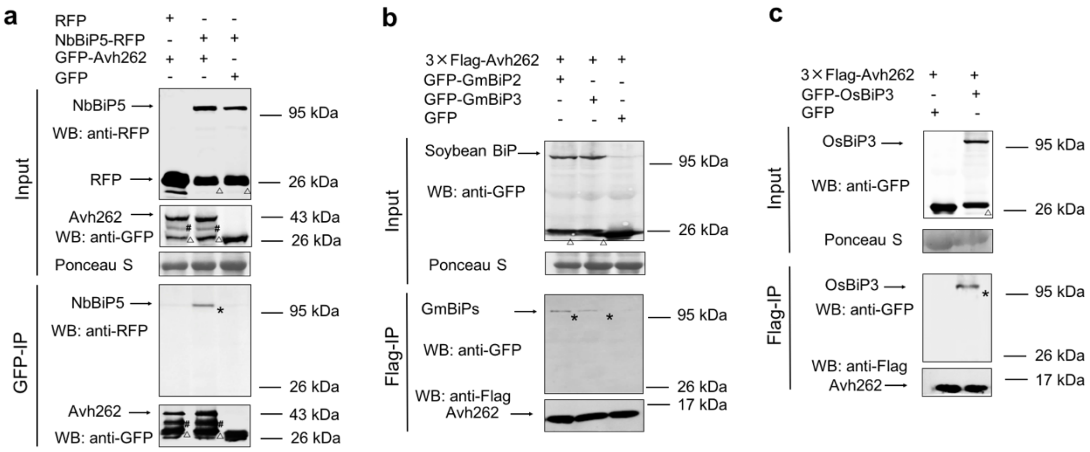

Supplementary Fig. 6. PsAvh262 interacts with NbBiP5, GmBiP2, GmBiP3 and OsBiP3.

(a) PsAvh262 interacts with NbBiP5 *in planta*. Total proteins were extracted from *N. benthamiana* leaves expressing GFP-PsAvh262 and NbBiP5-RFP. The protein complexes were pulled down using anti-GFP agarose beads. Co-precipitation of NbBiP5-RFP with GFP-PsAvh262 was detected by western blotting using anti-RFP antibody. (b) GmBiP2 and GmBiP3 co-immunoprecipitated with PsAvh262 *in planta*. GFP-GmBiP2, GFP-GmBiP3 or GFP were transiently expressed in *N. benthamiana* together with 3×Flag-PsAvh262. Co-precipitation of GFP-GmBiP2, GFP-GmBiP3 with PsAvh262 using anti-Flag antibodies was detected by western blotting using anti-GFP antibodies. (c) GFP-OsBiP3 co-immunoprecipitated with 3×Flag-PsAvh262 *in planta*. GFP-OsBiP3 or GFP was co-expressed with 3×Flag-PsAvh262 in *N. benthamiana*. Co-precipitation of GFP-OsBiP3 with PsAvh262 using anti-Flag antibodies was detected by western blotting using anti-GFP antibodies. Similar results were obtained in at least three independent experiments. In (a-c) GFP was attached to the N-termini of the BiP signal peptides, while RFP was attached to the C-terminus of the NbBiP5 HDEL motif. The N-terminus of Avh262 lacking its signal peptide was tagged with GFP (a) or 3xFlag (b,c). *, the objective bands of BiPs. △, non-specific band when using anti-GFP or anti-RFP; #, PsAvh262 derivatives band.

Supplementary Fig. 7

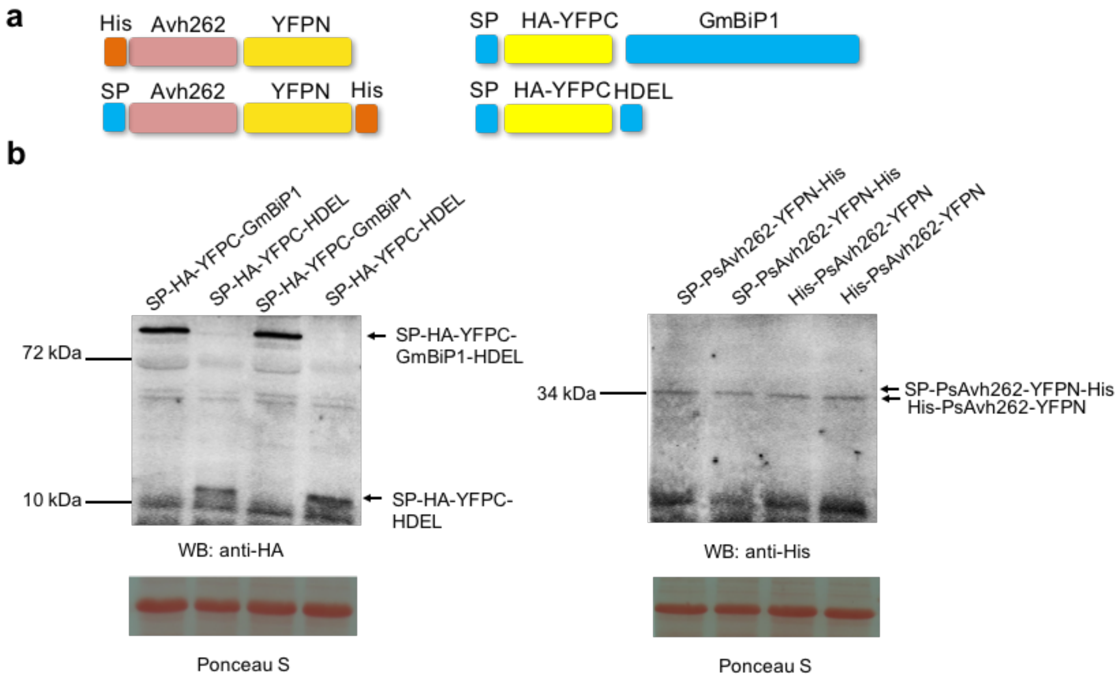

Supplementary Fig. 7. Structure and expression of constructs used for bimolecular fluorescence complementation (Related to Figure 4f, g).

(a) Schematic representation of expressed fusion proteins used in the bimolecular fluorescence complementation analysis (BiFC). SP, the signal peptide from the GmBiP1. HDEL, the ER retention signal of GmBiP1. (b) Immunoblots showing stability and full length of His-PsAvh262-YFPN, SP-PsAvh262-YFPN-His, SP-HA-YFPC-GmBiP1 and SP-HA-YFPC-HDEL fusions. These experiments were repeated three times with similar results.

Supplementary Fig. 8

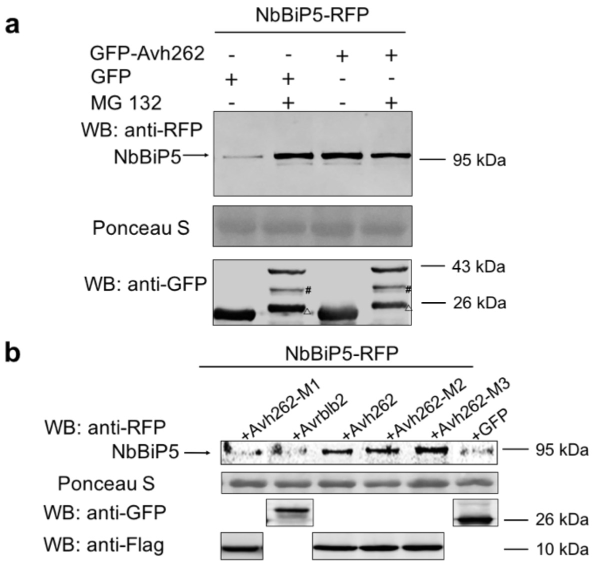

Supplementary Fig. 8. NbBiP5 can be stabilized by PsAvh262 or MG132.

(a) Immunoblots showing that the accumulation of NbBiP5-RFP (RFP was attached to the C-terminus of the HDEL motif) was enhanced by the expression of GFP-PsAvh262 or treatment with MG132 in *N. benthamiana*. (b) Immunoblots showing that the stabilization of NbBiP5-RFP by PsAvh262, PsAvh262-M2, or PsAvh262-M3, but not PsAvh262-M1, Avrblb2 or GFP. △, non-specific band when using anti-GFP; #, PsAvh262 derivatives band.

Supplementary Fig. 9

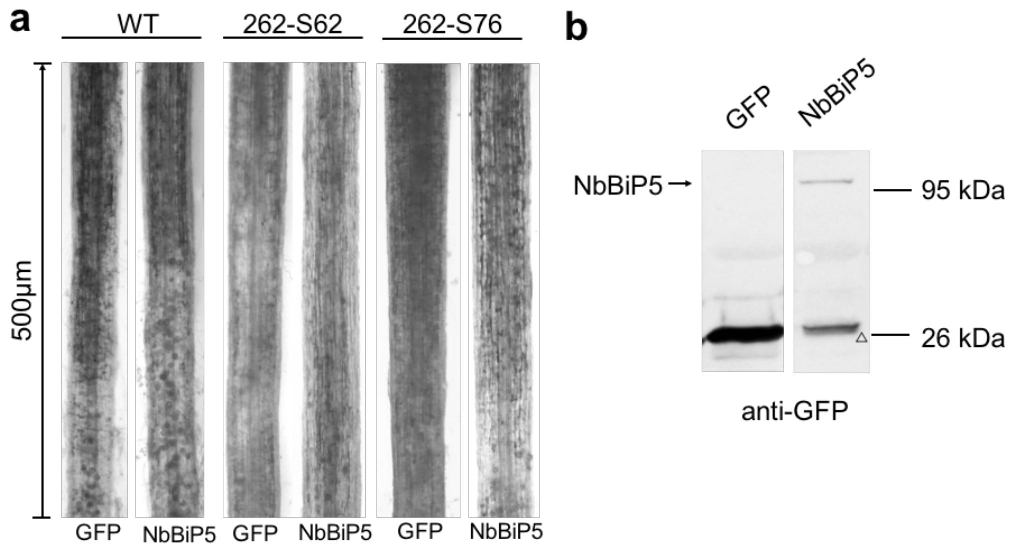

Supplementary Fig. 9. Overexpression of NbBiP5 in soybean can partly restore susceptibility to *PsAvh262*-silenced *P. sojae* transformants (Related to Fig. 8d). (a) Soybean hairy roots expressing GFP-SP-NbBiP5-HDEL or GFP were inoculated by wild-type *P. sojae* or *PsAvh262*-silenced *P. sojae* transformants (S62 and S76). Increased numbers of oospores developed in roots expressing NbBiP5 as shown by microscopic images at 48 hours after infection by mycelia of *PsAvh262*-silenced *P. sojae* transformants. Quantitation is shown in Fig. 8d. (b) The expression of *NbBiP5* was confirmed by western blotting. △, non-specific band when using anti-GFP.

Supplementary Fig. 10

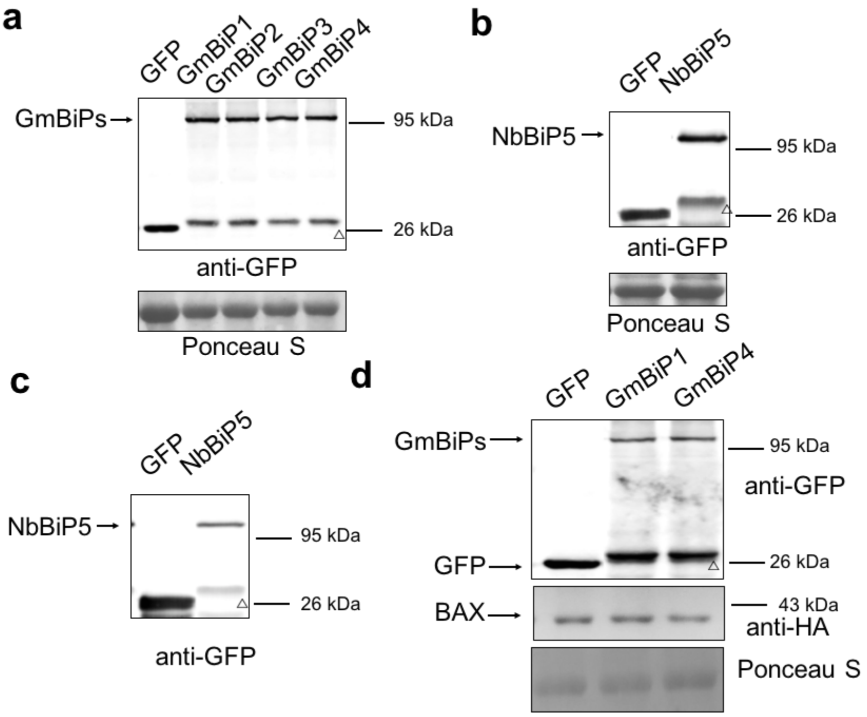

Supplementary Fig. 10. Immunoblots confirmed the expression of GFP-GmBiPs *in planta* (Related to Fig. 7).

Expression of GmBiPs (a) and NbBiP5 (b) in *N. benthamiana* were confirmed by western blotting. (c) The expression of NbBiP5 in soybean hairy roots was confirmed by western blotting. (d) Immunoblots confirmed the expression of GmBiP1 and GmBiP4 in *N. benthamiana*. In (a-d) GFP was fused to the N-terminus of the BiP signal peptides in each case. △, non-specific band when using anti-GFP.

Supplementary Fig. 11

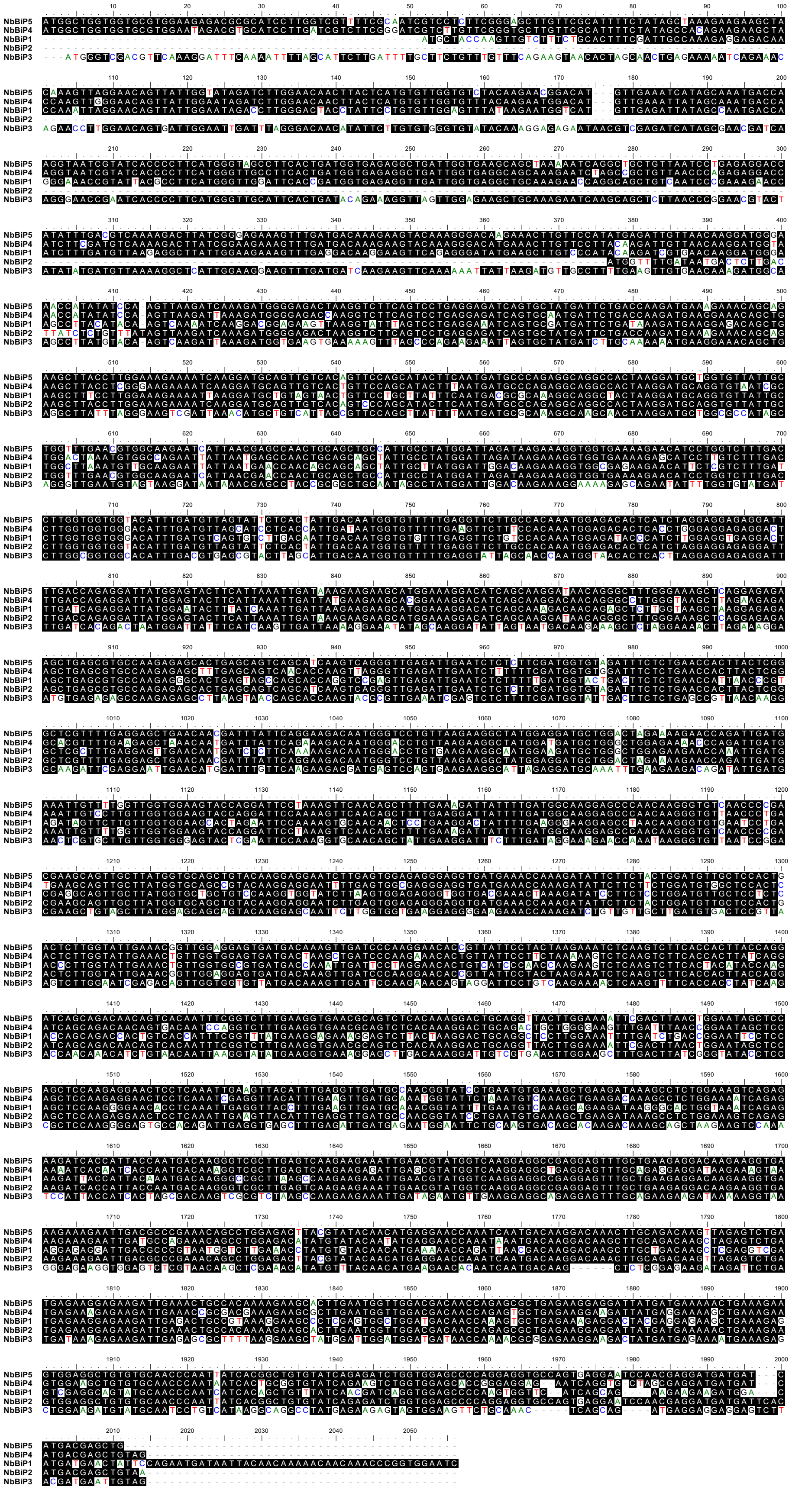

Supplementary Fig. 11. Nucleotide sequences of *NbBiPs* are highly conserved.

The multiple sequence alignment was generated with ClustalW (http://www.ch.embnet.org/software/ClustalW.html). Identical residues are colored by black regions.

Supplementary Fig. 12

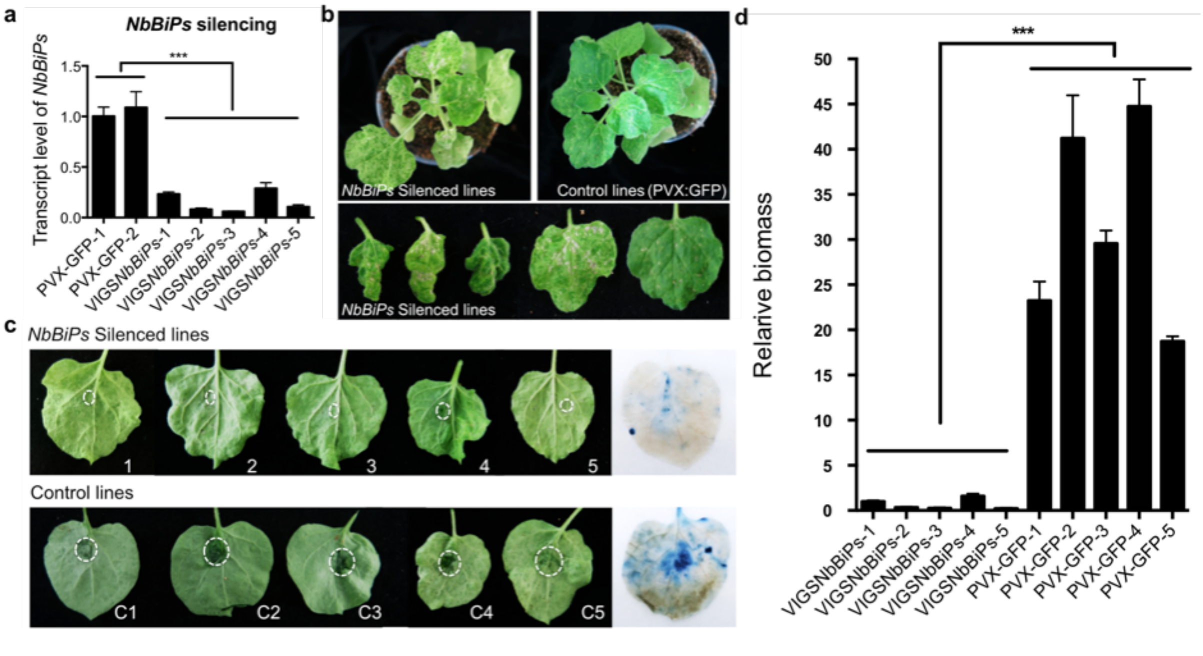

Supplementary Fig. 12. Silencing of *NbBiPs* in *N. benthamiana* enhanced plant resistance against *P. capsici.*

(a) qRT-PCR confirmed the silencing of *NbBiPs* in different plants. *NbBiPs* were silenced by potato virus X (PVX)-based virus-induced gene silencing (VIGS) constructs (pGR107:*NbBiPs*) in *N. benthamiana*. Error bars represent the mean ± s.d.(n=3), and asterisks (***) denote significant differences (*P*<0.001). (b) Silencing of *NbBiPs* in *N. benthamiana* led to cell death. The images were taken at 20 days post-infiltration of the silencing constructs. (c) Silencing of *NbBiPs* in *N. benthamiana* enhanced resistance to *P. capsici. NbBiP*-silenced and unsilenced leaves were inoculated with 100 zoospores of *P. capsici* at 20 days post-infiltration of the silencing constructs. The images were taken at 36 hpi. (d) Relative biomass of *P. capsici* determined by qPCR by measuring the ratio of *P. capsici* and *N. benthamiana* DNA at 36 hpi usning primers for the *actin* genes of each organism. PVX-GFP-1 to PVX-GFP-5 is control plants infiltrated with the VIGS vector carrying *GFP*. Error bars represent the mean ± s.d.(n=3), and asterisks (***) denote significant differences (*P*<0.001, one-way ANOVA) between samples.

Supplementary Fig. 13

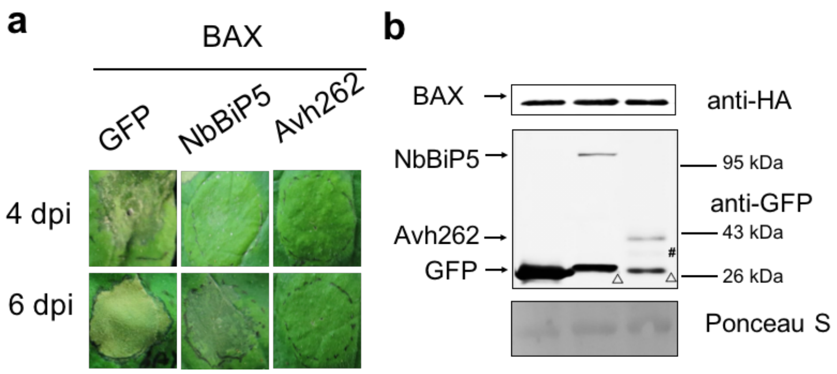

Supplementary Fig. 13. NbBiP5 overexpression attenuates BAX-triggered cell death.

(a) GFP-SP-NbBiP5-HDEL was expressed by agroinfiltration in *N. benthamiana* 24 hours before cells carrying *HA-BAX* were infiltrated. Leaves were photographed 4 and 6 days after *BAX* agro-infiltration. (b) Western blots probed with anti-GFP and anti-HA antibodies show that GFP-SP-NbBiP5-HDEL, GFP, GFP-PsAvh262 and BAX-HA were expressed in the infiltrated leaves. Similar phenotypes were observed in at least three independent experiments. △, non-specific band when using anti-GFP; #, PsAvh262 derivatives band.

Supplementary Fig. 14

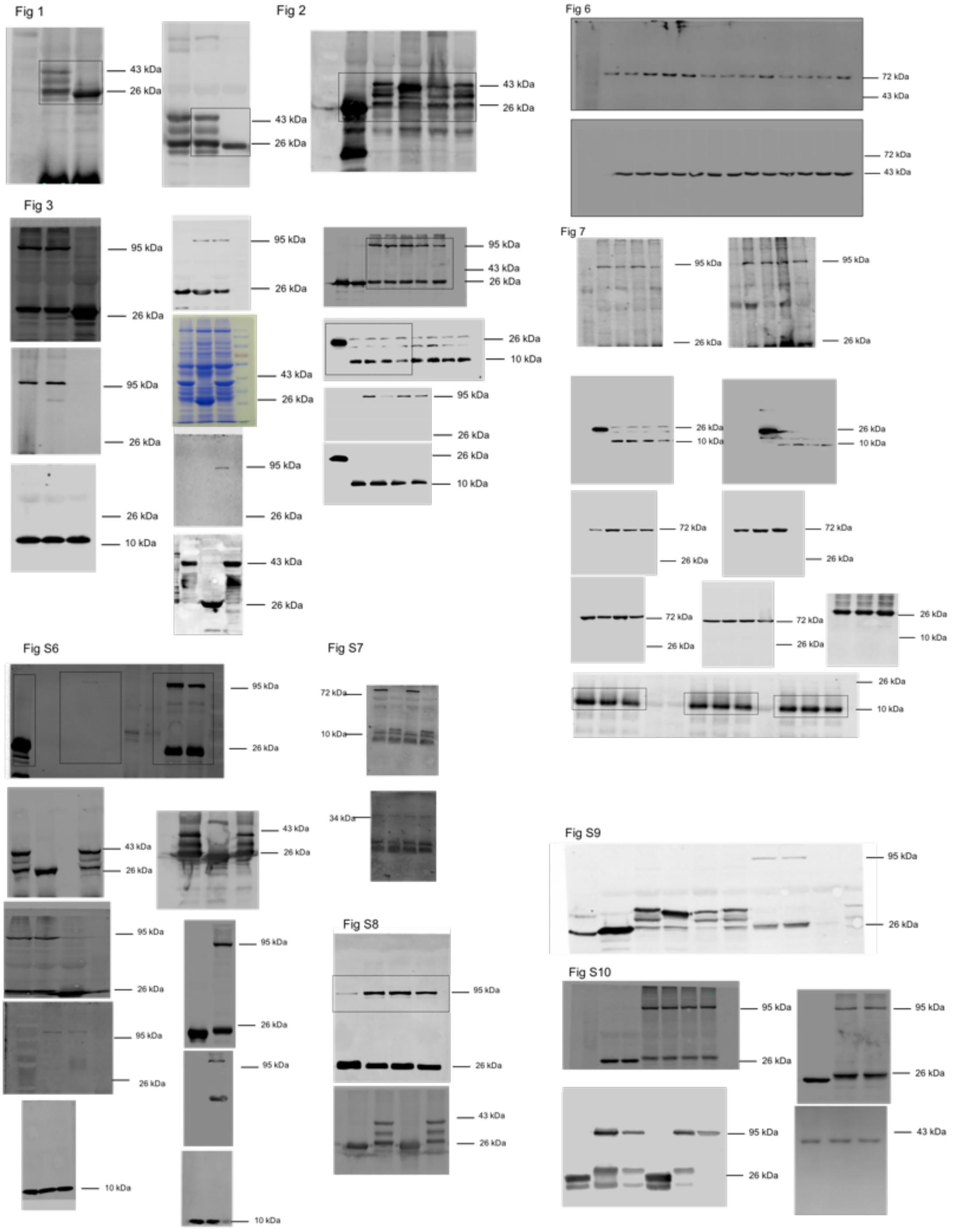

Supplementary Fig. 14. Uncropped scans of the blots and Coomassie Blue-stained gels used to generate the main manuscript figures. Molecular weight markers are indicated.

Supplementary Table 1

Accession	Description	No. of unique peptides
gi|729623	BiP 5 [*Nicotiana tabacum*]	4
gi|38325815	Heat shock protein 70-3 [*Nicotiana tabacum*]	7
gi|392465167	Heat shock protein 70 [*Nicotiana tabacum*]	6
gi|1732247	Transcription factor Myb1 [*Nicotiana tabacum*]	2
gi|19715913	Polyubiquitin-like protein [*Nicotiana tabacum*]	7
gi|11967906	Alpha tubulin [*Nicotiana tabacum*]	3
gi|436478	Salicylic acid binding catalase, partial [*Nicotiana tabacum*]	2
gi|2459684	Catalase 1 *[Nicotiana tabacum*]	2

Accession: GenBank accession no.

Supplementary Table 1. PsAvh262-associating proteins detected by co-immunoprecipitation (Co-IP) followed by liquid chromatography-tandem mass spectrometry (LC-MS/MS). Accession: GenBank accession no

Supplementary Table 2

**Primer name**	**Primer sequence (5'→3')**
qGmCYP2-F	CGGGACCAGTGTGCTTCTTCA
qGmCYP2-R	CCCCTCCACTACAAAGGCTCG
qGmPDI-F	TTGGTTGAAGGCGTACAAGGATGG
qGmPDI-R	ACTCCAGCAGAACATTCTTCCCAG
qGmVPE-F2	AACCCAAGGCCTGGAGTCAT
qGmVPE-R2	TCGCCGGTGTAATCCTTTG
RNAi-NbBiP5-F	CCGAGGAGTTTGCTGAAGAGG
RNAi-NbBiP5-R	CACCTCCTGGGGCTCCACCAG
qBip5i-F3	TTGACGCCCGAAACAGC
qBip5i-R3	GTCGTCCAACCATTCAAGT
qNbEF1a-F	TGGTGTCCTCAAGCCTGGTA
qNbEF1a-R	ACGCTTGAGATCCTTAACCGC
qPcAct-F	AGGAGATGGCCAAGTTAGC
qPcAct-R	CCGACTCATCATACTCGG
qPCR-GmBip-F	AGAAGGCTATGGAAGATGC
qPCR-GmBip-R	GCCACATCCAGGAGAAGG

qPCR262-F	ATGCTCCCGGTGGCCGTGGTCT
qPCR262-R	GACCTCCTTGACGACCTCCTTC
IF-PB-GmBip1-F	TACAAGGGTACCCCCATGGCTGGCTCGTGGGCACGCCGTTCTCTGAT
IF-PB-GmBip1-R	GGATCCGTCGACCCCGAGCTCTCGTGAGAATCATCCTCGTCT
IF-PB-GmBip2-F	TACAAGGGTACCCCCATGGCTTGCTCGTTTTCTCGCGGGTCTCTG
IF-PB-GmBip2-R	GGATCCGTCGACCCCGAGCTCGTCATGAGAATCTTCATCGTCGTCC
IF-PB-GmBip3-F	TACAAGGGTACCCCCATGGCTCGCTCGTTTTCACGCGGGTCTCTGCTTC
IF-PB-GmBip3-R	GGATCCGTCGACCCCGAGCTCGTCATGAGAATCATCATCCTCCTC
IF-PB-GmBip4-F	TACAAGGGTACCCCCATGGCTGGCTCGTGGGCACGCCGTTCTCTGATTGTTCTGGCTAT
IF-PB-GmBip4-R	GGATCCGTCGACCCCGAGCTCATCGTGAGAATCGTCCTCGTCTT
IF-PSRFP-GmBip4-F	CTTCTGCAGGGGCCCATGGCTGGCTCGTGGGCACGCCGTTCTCTGATTGTTCTGG
IF-PSRFP-GmBip4-R	GGCCATGTCGACCCCGAGCTCATCGTGAGAATCGTCCTCGTCTT
IF-PSRFP-GmBip1-F	CTTCTGCAGGGGCCCATGGCTGGCTCGTGGGCACGCCGTTCTCTGAT
IF-PSRFP-GmBip1-R	GGCCATGTCGACCCCGAGCTCATCGTGAGAATCATCCTCGTCTTCGCC

IF-PB-OsBiP3-F	TACAAGGGTACCCCCATGGATCGGGTTCGCGGATGCGCG
IF-PB-OsBiP3-R	GGATCCGTCGACCCCCTACAGCTCGTCATGCTCGTCGTCGAC
Avh262-SmaI-F	GGGatgGAAAGTATCCACCAGGTCAAC
Avh262-SmaI-R	GGGACGAACACCACCAGCACCGC
Avh262-KpnI-F	CGGGGTACCatgGAAAGTATCCACCAGGTCAAC
Avh262-BamHI-R	CGCGGATCCACGAACACCACCAGCACCGC
Avh262rev-XbaI-F	GCTCTAGAGAAAGTATCCACCAGGTCAAC
Avh262rev-ClaI-R	CATCGATACGAACACCACCAGCACCGC
Avh262-KpnI-F	CGGGGTACCatgGAAAGTATCCACCAGGTCAAC
Avh262-SacI-R	GCGAGCTCACGAACACCACCAGCACCGC
NbBIP5-IF-F	TACAAGGGTACCCCCATGGCTGGTGGTGCGTGGAATAG
NbBIP5-IF-R	GGATCCGTCGACCCCCAGCTCGTCATGATCATCATC
RNAi-NbBiPs-F	CCGAGGAGTTTGCTGAAGAGG
RNAi-NbBiPs-R	CACCTCCTGGGGCTCCACCAG
PVX-F	CAATCACAGTGTTGGCTTGC
PVX-R	GACCCTATGGGCTGTGTTG
pBinGFP2-F	AAAGACCCCAACGAGAAG

pBinGFP2-R	AACCCTAATTCCCTTATCT
pTOR-F	AGGCTCATTCTCCTTTTCACTC
pTOR-R	GGCCTTCTTTTGAAAACAATCG
pCAM1300-F	ATCTTCCCAAATTACCAATAC
pCAM1300-R	CAGGATTCAATCTTAAGAAAC
RFP-SalI-F	ACGCGTCGACATGGCCTCCTCCGAGGACGTC
RFP-KpnI-R	CGGGGTACCTTAGGCGCCGGTGGAGTGGCGG
PGEX-4T-2-F	GGGCTGGCAAGCCACGTTTGGTG
PGEX-4T-2-R	CCGGGAGCTGCATGTGTCAGAGG
PsACTIN-F	ACTGCACCTTCCAGACCATC
PsACTIN-R	CCACCACCTTGATCTTCATG

Supplementary Table 2. Primers used in this study.
